# Supplementary material for: WiFo-2: a generalist foundation model unifies heterogeneous wireless system design
Source: Natl Sci Rev. 2026 Aug 17;13(18):nwag500. doi: 10.1093/nsr/nwag500 (PMC13591638; doi:10.1093/nsr/nwag500)
Supplement: Supplementary file 1 [file nwag500_supplemental_file.pdf]

Supplementary information for **WiFo-2: a generalist foundation model unifies heterogeneous wireless system design**

Boxun Liu[1], Xuanyu Liu[1], Shijian Gao[2], Xuesong Cai[1], Xiang Cheng[1*], Liuqing Yang[2,3]

[1*]State Key Laboratory of Photonics and Communications, School of Electronics, Peking University, Beijing, 100871, China.
[2]Internet of Things Thrust, The Hong Kong University of Science and Technology (Guangzhou), Guangzhou, 511400, China.
[3]Intelligent Transportation Thrust, The Hong Kong University of Science and Technology (Guangzhou), Guangzhou, 511400, China.

*Corresponding author(s). E-mail(s): xiangcheng@pku.edu.cn;

Contents

1 Supplementary Note 1. Comparison with prior works 3

2 Supplementary Note 2. System model and problem description 6

3 Supplementary Note 3. Details of the LH-CSI dataset 9

4 Supplementary Note 4. Details of the model architecture 12

5 Supplementary Note 5. Details of pretraining scheme and applications 17

6 Supplementary Note 6. Details of network and pretraining experiment settings 19

7 Supplementary Note 7. Dataset, baselines, and implementation details for evaluation 20

8 Supplementary Note 8. Results on in-distribution channel reconstruction performance 26

9 Supplementary Note 9. Additional results for zero-shot channel reconstruction 26

10 Supplementary Note 10. Additional results for confidence prediction 33

11 Supplementary Note 11. Additional results for CSI-related downstream tasks 35

12 Supplementary Note 12. Visualization of the expert activation 36

13 Supplementary Note 13. Visualization of self-attention heat maps on different channel reconstruction tasks 36

14 Supplementary Note 14. Additional results for ablation studies 37

15 Supplementary Note 15. Additional results for scaling analysis 39

16 Supplementary Note 16. Implementation details of the over-the-air experiments 41

1 Supplementary Note 1. Comparison with prior works

This section reviews related studies on wireless foundation models and provides a comprehensive comparison with the proposed WiFo-2. According to whether the pretraining objective is explicitly tailored to specific physical-layer tasks, existing methods can be broadly categorized into two groups. The first group aims to learn task-agnostic or weakly task-related general wireless representations, which can be adapted to diverse wireless communication and sensing tasks through fine-tuning. The second group seeks to enhance the zero-shot capability of models on specific wireless tasks through dedicated pretraining strategies and network designs, thereby reducing or even eliminating the fine-tuning cost in target scenarios.

Task-agnostic general wireless representations.

SWiT [1] is among the early studies that introduced Transformers and self-supervised learning into wireless localization. It adopts a non-contrastive joint-embedding pretraining framework and learns wireless representations that are robust to fading and system perturbations through random channel augmentation. This method mainly supports wireless localization and demonstrates favourable transfer performance under few-shot fine-tuning and linear evaluation settings.

LWM [2] employs a Transformer encoder and masked channel modelling for large-scale wireless channel pretraining to learn task-agnostic channel embeddings. The model mainly enables Sub-6 GHz-to-mmWave beam prediction and LoS/NLoS classification through frozen representations or lightweight fine-tuning.

WirelessGPT [3] targets integrated sensing and communication scenarios by pretraining unified space–time–frequency wireless representations on large-scale wireless data. Its downstream tasks cover channel estimation, channel prediction, human activity recognition, and wireless/environment reconstruction, demonstrating the potential of general wireless representations in multi-task scenarios.

LWLM [4] is a wireless foundation model designed for localization. It adopts a Transformer architecture and incorporates spatial-frequency masked channel modelling, domain-transformation invariance, and position-invariant contrastive learning for pretraining. This method mainly supports ToA estimation, AoA estimation, single-BS localization, and multi-BS localization, and exhibits strong localization generalization under label-limited fine-tuning.

Enabling wireless task-aware capability.

WiFo [5] is the first study that explicitly enhanced zero-shot capabilities for wireless physical-layer tasks. It models CSI as a three-dimensional space–time–frequency tensor and adopts an MAE-based architecture with random, temporal, and frequency-masked reconstruction for pretraining. This method was the first to unify time-domain and frequency-domain channel prediction, and demonstrated the feasibility of performing physical-layer tasks without fine-tuning in target scenarios.

BERT4MIMO [6] adopts a BERT-like Transformer architecture to process massive MIMO CSI and learns channel representations through masked CSI reconstruction. This method mainly targets CSI reconstruction and prediction, and validates the effectiveness of masked modelling for MIMO CSI recovery and prediction across different mobility levels and channel scenarios.

ContraWiMAE [7] further introduces contrastive learning into a masked autoencoder framework, constructing augmented views based on wireless-channel characteristics such as noise, fading, and partial observations. This method supports cross-frequency beam selection, LoS detection, and channel estimation, while improving the discriminability and data efficiency of the learned representations.

WiFo-CF [8] extends task-aware wireless foundation models to FDD massive MIMO CSI feedback by designing a multi-user scalable autoencoder, dynamic-rate feedback, and a mixture-of-experts architecture. This method can handle CSI compression feedback under varying channel dimensions, feedback rates, user distributions, and data distributions, and demonstrates both zero-shot and few-shot generalization capabilities.

MUSE-FM [9] explores wireless foundation models from the perspective of multi-task physical-layer transceivers. It adopts an environment-aware, prompt-guided unified encoder–decoder and a hypernetwork to unify the input and output formats of different tasks. It covers tasks such as channel estimation, MIMO detection, channel decoding, multi-user precoding, and user localization, emphasizing unified modelling capability in multi-task wireless systems.

Overall, existing studies have progressed from general wireless representation learning to task-aware wireless foundation models. Nevertheless, several limitations remain.

- First, existing datasets are typically restricted to CSI from a single source, with fixed dimensions and system configurations, which severely limits the generalization ceiling of wireless foundation models across scenarios and configurations. The largest existing STF CSI dataset contains 3.3 billion CSI points from 19 heterogeneous subsets.
- Second, existing studies have paid limited attention to designing dedicated network architectures and pretraining methods for heterogeneous CSI data distributions, and the number of supported tasks remains very limited. Existing works support at most five few-shot fine-tuning tasks and at most two zero-shot tasks.
- Third, existing studies have not considered the additional reliability constraints of wireless communication systems or confidence estimation for model outputs. Moreover, few studies have explored inference-efficient model architectures, which limits their deployment in real-time wireless systems.
- Fourth, existing studies have been validated only through simulations, without constructing real hardware systems to verify their feasibility under practical communication conditions.

Compared with prior work, WiFo-2 makes the following advances.

- First, WiFo-2 constructs the world's largest open-source heterogeneous three-dimensional space–time–frequency CSI dataset, LH-CSI, which comprises 11.6

billion CSI points collected from 78 subsets. LH-CSI includes eight CSI data sources and covers all three acquisition modalities, namely statistical channel modelling, real-world measurements, and ray-tracing.

- Second, WiFo-2 designs an SMoE-based MDAE architecture that effectively handles both task and data heterogeneity. It also supports the largest number of wireless communication and sensing tasks to date, covering 12 tasks in total, including nine few-shot fine-tuning tasks and three zero-shot tasks.
- Third, WiFo-2 can accurately output confidence estimates for zero-shot reconstruction results, substantially enhancing its reliability for practical system deployment.
- Fourth, WiFo-2 builds the first hardware prototype empowered by a wireless foundation model for over-the-air experiments, demonstrating its performance advantages across three practical communication and sensing tasks in real-world environments under stringent latency and reliability requirements.

Finally, a comprehensive comparison between WiFo-2 and prior work is provided in Tables S1 and S2. The number of CSI points is computed as $\sum_{i=1}^{M} N_i K_i T_i$, where M is the total number of CSI samples, and N_i, K_i, and T_i denote the numbers of antennas, subcarriers, and temporal sampling points of the i-th CSI sample, respectively.

Table S1 Comprehensive comparison between WiFo-2 and prior works. Heterogeneous CSI indicates whether the model considers CSI with varying dimensions.

Study	Network structure	Pretraining strategy	Hetero-geneous CSI	Tasks via few-shot fine-tuning	Tasks via zero-shot inference	Confidence output	Hardware prototype
**SWIT** [1]	Transformer	joint-embedding self-supervised learning	×	1	0	×	×
**LWM** [2]	Transformer	Masked reconstruction	×	2	0	×	×
**WiFo** [5]	MAE	Masked reconstruction	✓	0	2	×	×
**BERT4MIMO** [6]	BERT-like Transformer	Masked reconstruction	×	0	1	×	×
**WirelessGPT** [3]	Transformer	Masked reconstruction	×	4	0	×	×
**ContraWiMAE** [7]	MAE	Masked reconstruction + contrastive learning	×	3	1	×	×
**LWLM** [4]	Transformer	Masked reconstruction + contrastive learning	×	4	0	×	×
**WiFo-CF** [8]	MUAE+MoE	Compressed reconstruction	✓	2	1	×	×
**MUSE-FM** [9]	Transformer	Supervised pretraining	✓	5	1	×	×
**WiFo-2**	**MDAE+SMoE**	**Two-stage mixed self-supervised pretraining**	✓	**9**	**3**	✓	✓

Table S2 Comparison of CSI datasets in WiFo-2 and prior work. S, T, and F denote the space, time, and frequency domains, respectively.

Study	CSI dimensions	Number of CSI points	Data sources	CSI acquisition method	Heterogeneous subsets
**SWIT** [1]	SF (2D)	0.5 B	1	Real-world measurement	1
**LWM** [2]	SF (2D)	1.0 B	1	Ray-tracing	1
**WiFo** [5]	STF (3D)	3.3 B	1	Statistical channel modeling	19
**BERT4MIMO** [6]	SF (2D)	0.1 B	1	Statistical channel modeling	1
**WirelessGPT** [3]	STF (3D)	/	3	Ray-tracing	/
**ContraWiMAE** [7]	SF (2D)	2.56 B	1	Ray-tracing	1
**LWLM** [4]	SF (2D)	24.0 B	1	Ray-tracing	1
**WiFo-CF** [8]	SF (2D)	4.1 B	5	Statistical channel modeling<br>Ray-tracing<br>Real-world measurements	32
**MUSE-FM** [9]	SF (2D)	1.6 B	1	Ray-tracing	1
**WiFo-2**	STF (3D)	11.6 B	8	Statistical channel modeling<br>Ray-tracing<br>Real-world measurements	78

2 Supplementary Note 2. System model and problem description

Space-time-frequency channel model

We study a canonical multiple-input multiple-output (MIMO) and orthogonal frequency division multiplexing (OFDM) system where the transmitter uses a planar array and the receiver has a single antenna. The planar array contains $N = N_h N_v$ elements, with N_h and N_v along the horizontal and vertical axes. Under a widely adopted geometric channel model with P paths, the CSI between the transmitter and the receiver at time t and frequency f is modeled as

$$\boldsymbol{h}(t,f) = \sum_{p=1}^{P} \beta_p \boldsymbol{a}(\phi_p, \theta_p) e^{j2\pi(\nu_p t - \tau_p f)}, \tag{1}$$

where β_p, ν_p, and τ_p represent the complex path gain, Doppler frequency shift, and the delay of the p-th path. The UPA steering vector $\boldsymbol{a}(\phi_p, \theta_p)$ is parameterized by the azimuth angle ϕ_p and elevation angle θ_p. Assume that T time samples are collected uniformly with spacing Δt, and K subcarriers are uniformly spaced by Δf. The resulting space–time–frequency CSI forms a tensor $\boldsymbol{H} \in \mathbb{C}^{T\times K\times N}$ satisfying

$$\boldsymbol{H}_{i,k,1:N} = \boldsymbol{h}(t_i, f_k), \qquad i = 1,\dots,T,\ k = 1,\dots,K, \tag{2}$$

where $t_i = i\Delta t$ and $f_k = f_1 + (k-1)\Delta f$ denote the i-th time instant and the k-th subcarrier, respectively. Here f_1 denotes the frequency of the first subcarrier.

CSI reconstruction tasks

Channel estimation. It aims to reconstruct the complete 3D CSI from partial observations at pilot locations. In practice, communication systems adopt diverse pilot configurations, including block, comb and scattered pilots, which makes it difficult

to process them with a single network. To address this challenge, we employ a two-stage estimation scheme. First, a coarse estimate of the full CSI, $\hat{\boldsymbol{H}}^{\text{co}}$, is obtained by three-dimensional interpolation of the pilot-location estimates. Second, a denoising refinement mapping function f_{CE} maps $\hat{\boldsymbol{H}}^{\text{co}}$ to the final estimate $\hat{\boldsymbol{H}}^{\text{ce}}$. Accordingly, we model channel estimation task as minimizing the mean squared error (MSE) between the estimate and the ground truth $\boldsymbol{H}$:

$$\min \|\boldsymbol{H} - \hat{\boldsymbol{H}}^{\text{ce}}\|_F^2, \quad s.t. \quad \hat{\boldsymbol{H}}^{\text{ce}} = f_{\text{CE}}\left(\hat{\boldsymbol{H}}^{\text{co}}\right). \tag{3}$$

Here $\|\cdot\|_F$ is the Frobenius norm.

Time-domain channel prediction. It predicts the CSI over the next T_h time samples from the CSI over the preceding $T - T_h$ samples. Therefore, we formulate the time-domain channel prediction task as minimizing the MSE between the predicted CSI $\hat{\boldsymbol{H}}^{\text{TCP}}$ and the ground truth, expressed as

$$\min \|\boldsymbol{H}_{T-T_h+1:T,1:K,1:N} - \hat{\boldsymbol{H}}^{\text{TCP}}_{T-T_h+1:T,1:K,1:N}\|_F^2, \tag{4}$$

$$s.t. \quad \hat{\boldsymbol{H}}^{\text{TCP}}_{T-T_h+1:T,1:K,1:N} = f_{\text{TCP}}(\boldsymbol{H}_{1:T-T_h,1:K,1:N}), \tag{5}$$

where f_{TCP} represents the mapping function for time-domain channel prediction.

Frequency-domain channel prediction. Similar to the prediction in the time domain, we aim to predict the CSI over the last $K - K_u$ sub-carriers based on the CSI of the first K_u sub-carriers. The frequency-domain channel prediction is also modeled as the MSE minimization problem as

$$\min \|\boldsymbol{H}_{1:T,K_u+1:K,1:N} - \hat{\boldsymbol{H}}^{\text{FCP}}_{1:T,K_u+1:K,1:N}\|_F^2, \tag{6}$$

$$s.t. \quad \hat{\boldsymbol{H}}^{\text{FCP}}_{1:T,K_u+1:K,1:N} = f_{\text{FCP}}(\boldsymbol{H}_{1:T,1:K_u,1:N}), \tag{7}$$

where f_{FCP} is the mapping function in the frequency-domain and $\hat{\boldsymbol{H}}^{\text{FCP}}$ denotes the predicted CSI for the frequency domain.

It is observed that the above three tasks can be uniformly formulated as a CSI reconstruction task, which obtains complete and accurate CSI with partial or coarse estimated CSI. Denote the estimated partial CSI as $\hat{\boldsymbol{H}}_\Omega$, where Ω represents the subset of all elements. The unified channel reconstruction task can be modeled as

$$\min \|\boldsymbol{H} - \boldsymbol{H}^{\text{rec}}\|_F^2, \; s.t. \, \boldsymbol{H}^{\text{rec}} = f_{\text{rec}}(\hat{\boldsymbol{H}}_\Omega), \tag{8}$$

where f_{rec} represents the mapping function of the unified channel reconstruction and $\boldsymbol{H}^{\text{rec}}$ denotes the reconstructed CSI.

CSI-related downstream tasks

We evaluate 9 CSI-related downstream tasks to provide a comprehensive assessment of the enabling capability of the pretrained general-purpose representation across diverse applications.

Scenario Classification. This task aims to determine from CSI whether the link between the transmitter and the receiver contains a LoS path. This deepens understanding of the propagation environment and guides precoding and related strategy choices. The input is a three-dimensional CSI sample, and the output is a scene label, LoS or NLoS. The task evaluates the model's ability to extract multipath characteristics from CSI.

Sub-6 GHz to mmWave Beam Prediction. MmWave bands are critical for 5G and future 6G systems. Communication devices are typically equipped with antennas that cover both low-frequency (sub-6 GHz) and mmWave frequencies. This task uses sub-6 GHz CSI to predict the optimal mmWave beam, thereby reducing beam-sweeping overhead. The input is an instantaneous two-dimensional subcarrier-by-antenna CSI sample. The output is the codebook index of the optimal mmWave beam. The task assesses the model's capability for cross-frequency, geometry-aware alignment.

Wireless Localization. Wireless localization leverages CSI to estimate user position while preserving privacy and delivering precise location services. It serves as an important complement in environments where global navigation satellite systems (GNSS) are degraded or unavailable, especially indoors. The input is a two-dimensional subcarrier-by-antenna CSI sample of the communication link, and the output is the user's two-dimensional position coordinates. The task comprehensively assesses the model's ability to extract delay and angular information from raw CSI.

CSI Feedback. CSI feedback aims to compress and reconstruct channel state information efficiently so that accurate recovery is achieved with minimal overhead. Following [10], we cast the problem as channel denoising and apply post-processing to channels reconstructed by existing compression and feedback methods to further improve accuracy. The task requires general channel denoising capability and assesses the model's ability to extract generic channel characteristics.

AoA Estimation. AoA estimation aims to infer the angle of arrival (AoA) of the channel's dominant path from CSI, providing critical priors for subsequent beamforming and localization. The task takes as input a two-dimensional CSI sample at a given time instant and outputs the AoA of the current channel's dominant path. It assesses the model's spatial-spectrum super-resolution and dominant-path separability, and further evaluates its robust generalization in complex propagation environments.

Doppler Estimation. In communication scenarios such as satellite systems and high-speed railways, the extremely high mobility induces pronounced time variations in the wireless channel. Doppler estimation aims to accurately characterize and compensate for the rapid phase variations caused by high relative motion, thereby improving channel tracking accuracy and ensuring reliable signal demodulation and stable communication links. This task is designed to extract Doppler-shift information from three-dimensional CSI, thereby evaluating the model's ability to capture temporal variation patterns in wireless channels.

Cross-band channel prediction. In frequency-division duplexing (FDD) systems, the uplink and downlink work on non-adjacent frequency bands, which necessitates additional CSI feedback. Predicting downlink CSI from uplink CSI can reduce or

even eliminate downlink CSI feedback and thereby substantially improve spectral efficiency. Unlike frequency-domain channel prediction, this task seeks to predict CSI in one band from CSI observed in another, non-adjacent band, posing a stricter challenge for modeling the frequency-domain correlations of CSI.

Vision-aided Frequency-domain Channel Prediction. Communication devices such as smartphones and embodied robots typically integrate wireless transceivers and multi-modal sensing modules. Because both multi-modal sensory data and the wireless channel observe the same physical space, they exhibit inherent correlation [11]. This task leverages visual information from the environment to enhance frequency-domain channel prediction. Given a co-temporal pair comprising an instantaneous two-dimensional subcarrier-by-antenna CSI sample and an RGB image, the output is a frequency-domain CSI prediction. The task assesses the model's ability to leverage auxiliary side information to enhance channel prediction.

Signal detection. CSI is critical for accurately recovering the transmitted symbols at the receiver. Consider a link with an N_a-antenna transmitter and a single-antenna receiver. Let the transmit symbol be $x \in \mathbb{C}$, the spatial-domain channel be $\boldsymbol{h}_{\mathrm{s}} \in \mathbb{C}^{N_a}$, and the received signal be $\boldsymbol{y} = \boldsymbol{h}_{\mathrm{s}} x + \boldsymbol{n}$, where $\boldsymbol{n} \in \mathbb{C}^{N_a}$ denotes noise. The signal detection task aims to recover x from the observed $\boldsymbol{y}$ and the channel $\boldsymbol{h}_{\mathrm{s}}$. This task evaluates the model's spatial-domain detection and interference suppression under imperfect CSI.

3 Supplementary Note 3. Details of the LH-CSI dataset

Detailed descriptions of each source are provided below.

QuaDRiGa. The dataset was generated by the 3GPP-compliant generator QuaDRiGa [12] through statistical channel modeling, which allows for the flexible adjustment of various system configurations and thereby increases the dataset's diversity. The dataset includes 19 coarse-grained sub-datasets QC1 to QC19, and 19 fine-grained CSI sub-datasets QF1 to QF19, covering typical scenarios such as UMa, UMi, indoor, and RMa. It spans multiple 5G NR frequency bands from 1.5 GHz to 28 GHz, supports antenna arrays ranging from 4 to 32 elements, and accommodates user speeds ranging from 0 to 300 km/h.

PKU-GBSM. The dataset is self-developed based on a spatiotemporal-consistent and space-time-frequency triple non-stationary channel model [13], serving as a supplement to QuaDRiGa. It is designed for dynamic vehicle-to-vehicle (V2V) scenarios in vehicular networks and can effectively model continuous arbitrary trajectories of connected vehicles. The dataset includes one coarse-grained sub-dataset VC and one fine-grained CSI sub-dataset VF, with a frequency of 5.9 GHz and 16 antennas.

LuViRA. The dataset was constructed by processing the radio modality of the open-source LuViRA [14] dataset, which is a synchronized dataset spanning vision, radio, and audio for indoor localization research. LuViRA contains 89 trajectories that are either straight lines or random. For each trajectory, it provides three-dimensional CSI with 100 samples along the time, subcarrier, and antenna dimensions. Data were collected at 100 Hz with a 20 MHz transmission bandwidth using an irregular planar

antenna array, and the receiver moved at low speed. We continuously segmented the data into contiguous CSI samples of size $16\times16\times64$ along the time, antenna, subcarrier dimensions and randomly selected 12,000 samples to construct a coarse-grained CSI dataset LC.

DICHASUS. The dataset was constructed by processing the open-source massive MIMO CSI dataset DICHASUS [15], which provides channel measurements and position labels across a variety of indoor and outdoor scenarios. The coarse-grained CSI sub-datasets DC1, DC2, and DC3 are drawn from the indoor LoS dataset dichasus-015x, and from the two factory datasets dichasus-adxx and dichasus-cf0x, respectively. From the original 1024 subcarriers, we retained the first 512 and applied smoothing to obtain 64 subcarriers. In addition, we selected the first 64 antenna elements from the original 100-element array. Since the original CSI samples contain only the subcarrier and antenna dimensions, we replicated each sample four times along the time axis to construct 3D CSI.

Argos. The dataset was constructed by processing the open-source Argos [16] dataset, which contains many-antenna MU-MIMO channel measurements collected in indoor and outdoor scenarios. We used subsets 9, 34, 38, 39, 41, 43, and 56 to construct the coarse-grained CSI sub-datasets AC1 to AC7, respectively. The measurements operated at 2.4 GHz with 96 antennas. Although the raw data include periodic temporal sampling, the sampling interval is too large to support time-domain channel prediction. We therefore used only the two-dimensional subcarrier-by-antenna CSI at each time sample and augmented along the time dimension. In fact, this replication-based expansion along the temporal dimension is reasonable, as such a scenario can itself be regarded as a static-channel or extremely low-dynamics case. This expansion does not compromise the model's temporal modeling capability. Rather, it helps the model learn corner cases with slowly time-varying channel characteristics.

PKU-RM. Although existing publicly available CSI datasets are relatively large, their system configurations, such as frequency points, are limited. Additionally, the temporal sampling intervals are quite large, which makes it difficult to construct a fine-grained CSI dataset for channel estimation tasks. To address this, we developed a channel measurement platform based on a software-defined radio system (Ettus USRP X410) and conducted CSI measurements in dynamic indoor and outdoor environments on the Peking University campus to create the PKU-RM dataset. Both the transmitter and the receiver were equipped with a single rod antenna and synchronized using rubidium clocks. During data collection, the transmitter remained stationary, while the receiver was moved at a low speed by a person holding the antenna, thereby emulating wireless communication with a mobile terminal. During the measurements, the transmitter continuously transmitted Zadoff–Chu sequences of length 2048 at a sampling rate of 40 MHz. The receiver obtained the CIR through correlation and further derived the CSI on each subcarrier by applying an FFT. This dataset consists of 9 coarse-grained sub-datasets (RMC1 to RMC9) and 9 fine-grained sub-datasets (RMF1 to RMF9). For the fine-grained sub-datasets, the temporal sampling interval of the CSI is the inverse of the subcarrier spacing, in accordance with the time-frequency relationship of the resource block. The dataset covers 9 different frequency points and

various subcarrier spacing configurations. Since the measurement platform only supports a single-input single-output (SISO) link, we replicated each sample four times along the antenna dimension to construct 3D CSI. Similar to the temporal expansion used for the Argos dataset, the replication-based expansion along the antenna dimension here is also reasonable. Notably, wireless channels vary continuously in space. Therefore, this antenna-dimensional expansion can emulate scenarios with very small antenna spacing and helps enhance the model's channel modeling capability under single-antenna configurations.

DeepMIMO. Our dataset was constructed by processing DeepMIMO [17], an open-source ray-tracing dataset generated with Remcom's Wireless InSite. DeepMIMO allows flexible adjustment of system configurations and follows 5G NR time and frequency settings. We selected the O1_3p4, O1_28, and O1_drone_200 scenarios to simulate the coarse-grained datasets DEC1, DEC2, DEC3 and the fine-grained CSI datasets DEF1, DEF2, DEF3, respectively. The corresponding carrier frequencies are 3.4 GHz, 28 GHz, and 200 GHz, with diverse user velocities. During generation, the channel model for all three scenarios was set to CDL-D.

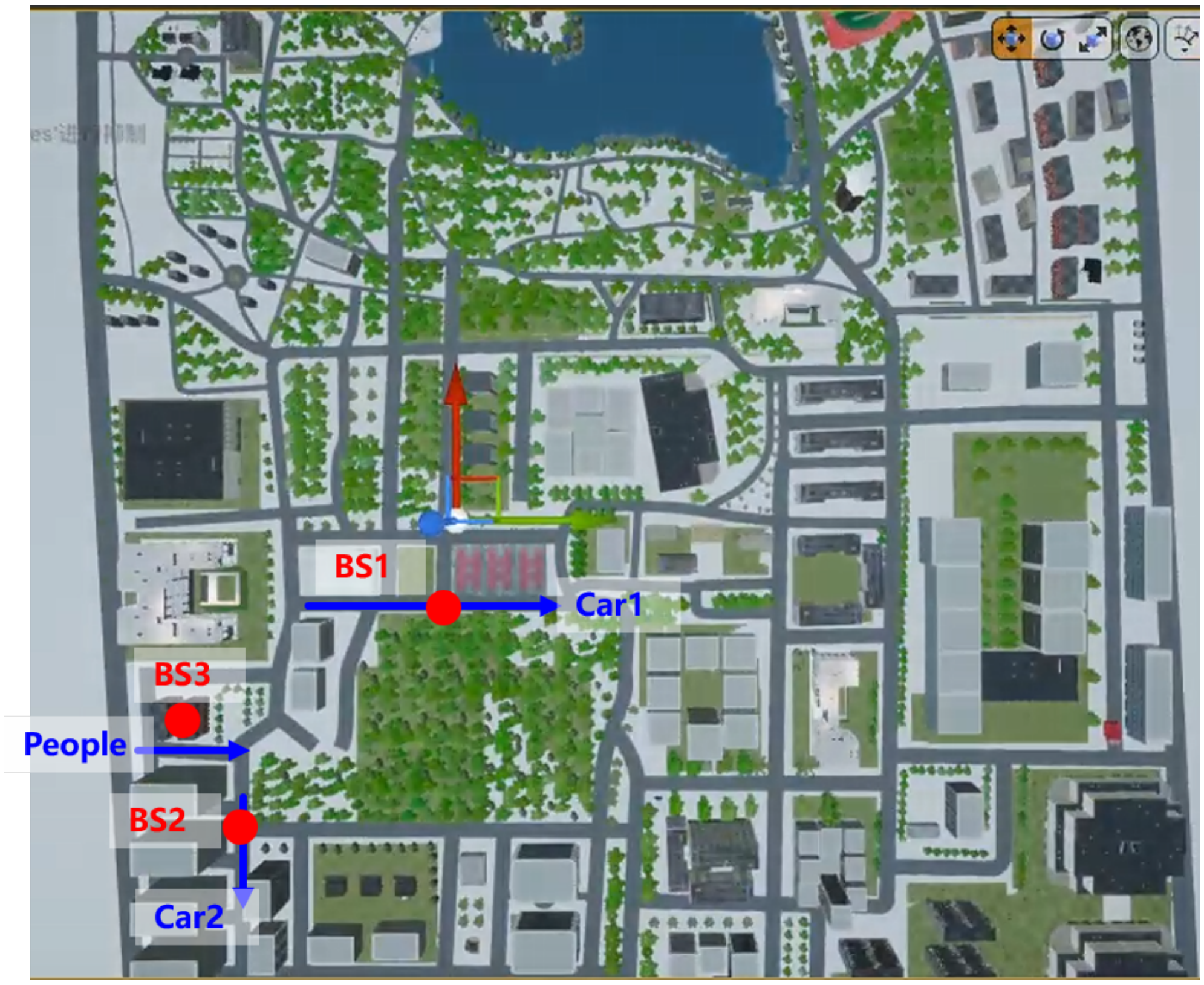

Fig. S1 Scenario illustration of the constructed SionnaRT-based PKU-RT dataset.

PKU-RT. Although DeepMIMO offers a rich set of scenarios, its scene modeling is relatively coarse, and the environments lack static scatterers, leading to scattering characteristics that differ markedly from reality. To address this, we constructed a simulated Peking University campus and performed ray tracing with SionnaRT [18], which is a lightning-fast stand-alone ray tracer for radio propagation modeling. As shown in Fig. S1, the scene is modeled at higher fidelity and includes dynamic scatterers such as vehicles and pedestrians. We built three coarse-grained CSI sub-datasets, RTC1, RTC2, and RTC3. Specifically, the communication link in the RTC1 dataset is selected between BS1 and Car1, the link in the RTC2 dataset is selected between BS2 and Car2, and the link in the RTC3 dataset is selected between BS3 and the moving people. The locations of the BSs, as well as the trajectories of the vehicles and people, are shown in Fig. S1. Vehicles and pedestrians move at low, nonuniform speeds, with a temporal sampling interval of 2 ms.

Then, the detailed system configurations of the constructed LH-CSI are illustrated, including the source, center frequency f_{C}, subcarrier number K and spacing Δf, time sample number T and interval Δt, antenna configuration, scenario, and user velocity of each sub-dataset. The datasets from the statistical channel modeling, real-world measurement, and ray-tracing parts are presented separately in Tables S3, S4, and S5, respectively. It can be observed that the constructed LH-CSI dataset exhibits pronounced heterogeneity in both system configurations and CSI distributions, providing strong support for WiFo's pretraining and generalization testing.

4 Supplementary Note 4. Details of the model architecture

As shown in Fig. S2, WiFo-2 adopts an SMoE-based MDAE architecture comprising a preprocessor, an encoder, and a decoder. The designed network architecture aims to enable WiFo-2 to learn general wireless representations while effectively handling heterogeneity across data and tasks. On the one hand, since electromagnetic fields vary continuously along the time, space, and frequency dimensions, there exists substantial redundancy in CSI. Therefore, the design of masked reconstruction or denoising forces the network to recover complete CSI from partial or corrupted observations, encouraging the model to learn the intrinsic sparse multipath structure of electromagnetic fields and thereby construct general wireless representations with strong generalization capability. On the other hand, different patches of a CSI tensor are inherently heterogeneous, and the same CSI patch may require different features for different tasks. Therefore, the SMoE design makes token processing dependent on both the data distribution and the task, effectively modeling these two types of heterogeneity and resolving the tension of using a single model to handle heterogeneous distributions and tasks. The detailed architecture is described as follows.

Preprocessor

To facilitate neural network processing, we convert the three-dimensional complex CSI tensor into $\boldsymbol{x} \in \mathbb{R}^{2\times T\times K\times N}$, where the two channels along the first dimension correspond to the real and imaginary parts of the original CSI. Here, T, K, and

Table S3 An illustration of the system configurations for the part of LH-CSI generated using statistical channel modeling.

Source	Dataset	f_C(GHz)	K	Δf(kHz)	T	Δt(ms)	Antenna	Scenario	User speed(km/h)
QuaDRiGa	QC1	1.5	128	60	32	1	1×4	UMi NLoS	3-50
	QC2	1.5	128	90	32	0.5	2×4	RMa NLoS	100-200
	QC3	1.5	64	90	16	1	1×8	Indoor LoS	0-10
	QC4	1.5	32	180	16	0.5	4×8	UMa LoS	30-100
	QC5	2.5	64	90	16	0.5	2×2	RMa LoS	100-200
	QC6	2.5	128	90	32	1	2×4	UMi LoS	3-50
	QC7	2.5	32	360	16	0.5	4×8	UMa LoS	30-100
	QC8	2.5	64	90	16	1	4×4	Indoor NLoS	0-10
	QC9	4.9	128	60	32	1	1×4	UMi NLoS	3-50
	QC10	4.9	64	180	16	0.5	2×4	UMa LoS	100-200
	QC11	4.9	64	60	16	0.5	4×4	UMa NLoS	30-100
	QC12	4.9	32	180	16	1	4×8	Indoor LoS	0-10
	QC13	5.9	64	90	16	0.5	2×8	UMa NLoS	100-200
	QC14	5.9	128	60	32	1	2×4	UMi NLoS	3-50
	QC15	5.9	64	90	16	1	4×4	Indoor +LoS	0-10
	QC16	5.9	32	360	16	0.5	4×8	UMa NLoS	30-100
	QC17	3.5	64	60	16	0.5	2×4	UMa NLoS	30-100
	QC18	6.7	64	60	16	1	4×4	UMi LoS	3-50
	QC19	28	64	360	16	0.5	2×8	UMa LoS	30-100
	QF1	1.5	72	60	28	0.017	1×4	UMi NLoS	3-50
	QF2	1.5	72	15	28	0.067	2×4	RMa NLoS	100-200
	QF3	1.5	72	15	28	0.067	1×8	Indoor LoS	0-10
	QF4	1.5	36	30	12	0.033	4×8	UMa LoS	30-100
	QF5	2.5	72	30	28	0.033	2×2	RMa LoS	100-200
	QF6	2.5	72	15	28	0.067	2×4	UMi LoS	3-50
	QF7	2.5	36	60	12	0.017	4×8	UMa LoS	30-100
	QF8	2.5	36	15	12	0.067	4×4	Indoor NLoS	0-10
	QF9	4.9	72	60	28	0.017	1×4	UMi NLoS	3-50
	QF10	4.9	72	30	28	0.033	2×4	UMa LoS	100-200
	QF11	4.9	36	60	12	0.017	4×4	UMa NLoS	30-100
	QF12	4.9	36	30	12	0.033	4×8	Indoor LoS	0-10
	QF13	5.9	36	15	12	0.067	2×8	UMa NLoS	100-200
	QF14	5.9	72	60	28	0.017	2×4	UMi NLoS	3-50
	QF15	5.9	36	15	12	0.067	4×4	Indoor LoS	0-10
	QF16	5.9	36	15	12	0.067	4×8	UMa NLoS	30-100
	QF17	3.5	72	15	12	0.067	2×4	UMa NLoS	30-100
	QF18	6.7	36	15	12	0.067	4×4	UMi LoS	3-50
	QF19	28	72	30	16	0.033	2×8	UMa LoS	30-100
PKU-GBSM	VC	5.9	64	360	16	1	4×4	V2V	108 & 36
	VF	5.9	72	60	12	0.017	2×4	V2V	108 & 36

Table S4 An illustration of the system configurations for the part of LH-CSI generated via real-world measurement.

Source	Dataset	f_C(GHz)	K	Δf(kHz)	T	Δt(ms)	Antenna	Scenario	User speed(km/h)
LuViRA	LC	3.7	64	200	16	10	64	Indoor	~ 0.5
DICHASUS	DC1	1.3	64	391	4	-	64	Indoor LoS	-
	DC2	3.4	64	391	4	-	64	Factory	-
	DC3	5.9	64	391	4	-	64	Factory	-
Argos	AC1	2.4	52	313	4	-	96	Indoor	-
	AC2	2.4	52	313	4	-	96	Indoor	-
	AC3	2.4	52	313	4	-	96	Outdoor	-
	AC4	2.4	52	313	4	-	96	Outdoor	-
	AC5	2.4	52	313	4	-	96	Outdoor	-
	AC6	2.4	52	313	4	-	96	Outdoor	-
	AC7	5.0	52	313	4	-	96	Outdoor	-
PKU-RM	RMC1	3.8	64	180	16	0.5	4	Indoor LoS	~ 2
	RMC2	4.6	64	180	16	0.5	4	Indoor NLoS	~ 2
	RMC3	3.3	64	90	16	1	4	Indoor NLoS	~ 2
	RMC4	2.0	64	90	16	1	4	Indoor LoS	~ 2
	RMC5	5.5	64	360	16	0.25	4	Indoor LoS	~ 2
	RMC6	1.6	64	180	16	0.5	4	Outdoor LoS	~ 5
	RMC7	2.1	64	180	16	0.5	4	Outdoor LoS	~ 5
	RMC8	3.7	64	180	16	0.5	4	Outdoor LoS	~ 5
	RMC9	5.2	64	180	16	0.5	4	Outdoor LoS	~ 5
	RMF1	3.8	72	30	28	0.033	4	Indoor LoS	~ 2
	RMF2	4.6	72	30	28	0.033	4	Indoor NLoS	~ 2
	RMF3	3.3	72	15	28	0.067	4	Indoor NLoS	~ 2
	RMF4	2.0	72	15	28	0.067	4	Indoor LoS	~ 2
	RMF5	5.5	72	60	28	0.017	4	Indoor LoS	~ 2
	RMF6	1.6	72	30	28	0.033	4	Outdoor LoS	~ 5
	RMF7	2.1	72	30	28	0.033	4	Outdoor LoS	~ 5
	RMF8	3.7	72	30	28	0.033	4	Outdoor LoS	~ 5
	RMF9	5.2	72	30	28	0.033	4	Outdoor LoS	~ 5

N denote the number of time samples, subcarriers, and antennas, respectively. To accommodate inputs with varying CSI sizes, we partition $\boldsymbol{x}$ into non-overlapping 3D patches of size $(p_\mathrm{t}, p_\mathrm{f}, p_\mathrm{s})$ along the time, frequency, and spatial axes, respectively. The patching operation facilitates modeling of local CSI structure and substantially reduces the number of tokens, thereby lowering computational cost. The generated $L = \left\lceil \frac{T}{p_t} \right\rceil \left\lceil \frac{K}{p_f} \right\rceil \left\lceil \frac{N}{p_a} \right\rceil$ patches are further mapped to input tokens $\boldsymbol{x}_\mathrm{e} \in \mathbb{R}^{L \times D}$ via a linear layer, where $\lceil \cdot \rceil$ is the ceiling operator and D is the hidden dimension. For masked reconstruction pretraining tasks, only the visible tokens are fed to the network.

Table S5 An illustration of the system configurations for the part of LH-CSI generated via ray-tracing.

Source	Dataset	f_C(GHz)	K	Δf(kHz)	T	Δt(ms)	Antenna	Scenario	User speed(km/h)
DeepMIMO	DEC1	3.4	64	120	16	0.233	8	Outdoor	$100-120$
	DEC2	28	32	480	16	0.029	4×8	Outdoor	$100-300$
	DEC3	200	64	960	16	0.029	2×8	Drone	$30-50$
	DEF1	3.4	72	30	28	0.022	8	Outdoor	$100-120$
	DEF2	28	36	120	12	0.008	4×8	Outdoor	$100-300$
	DEF3	200	72	120	12	0.008	2×8	Drone	$30-50$
PKU-RT	RTC1	5.9	64	180	16	2	8	V2I	~ 10
	RTC2	5.9	64	180	16	2	8	V2I	~ 10
	RTC3	5.9	64	180	16	2	8	Pedestrian	~ 5

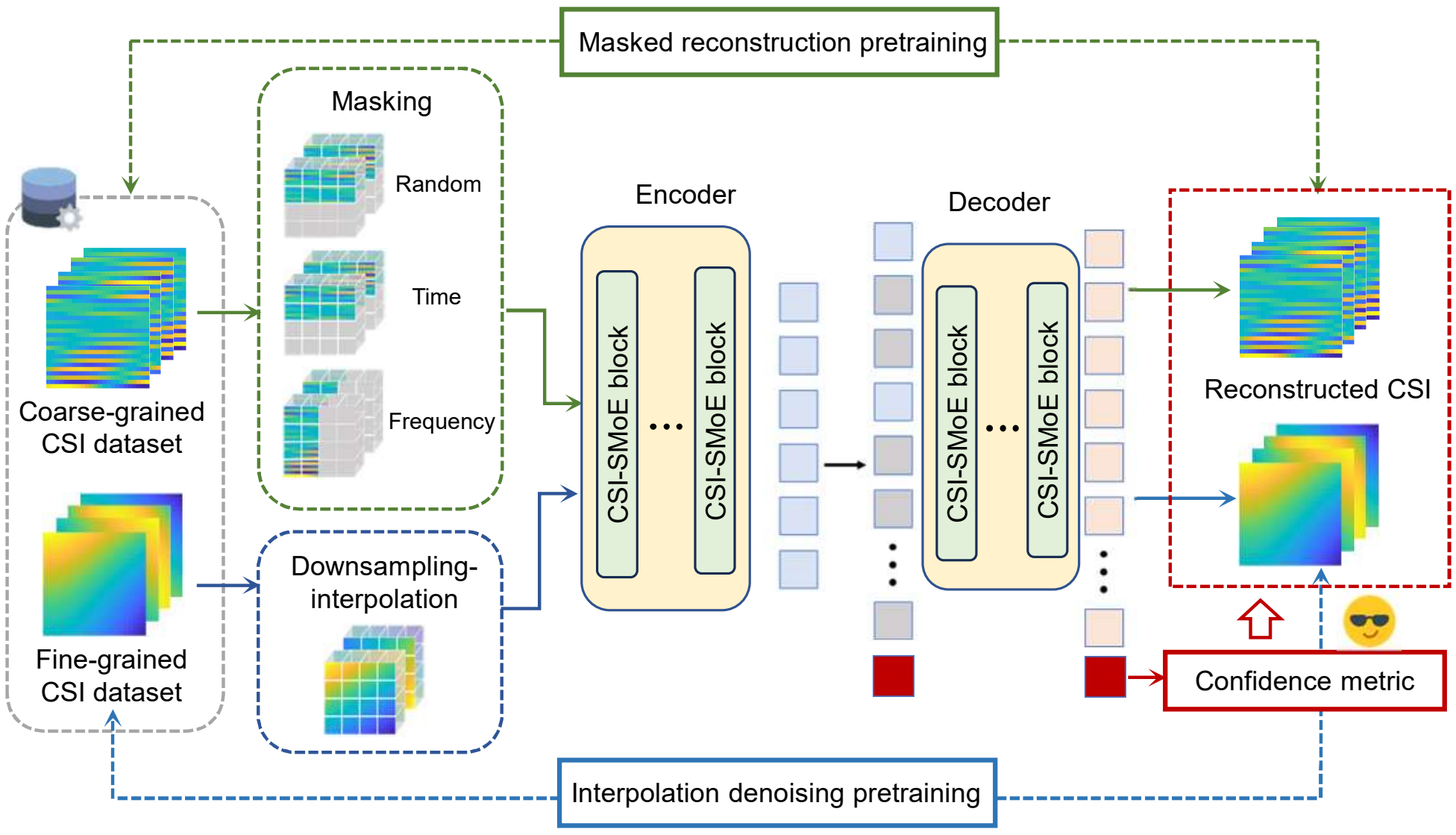

Fig. S2 Detailed illustration of WiFo-2 network architecture and pretraining mechanism.

The masking process is defined as

$$[\boldsymbol{x}_{\text{vis}}, \boldsymbol{x}_{\text{mask}}] = \text{MASK}(\boldsymbol{x}_{\text{e}}), \tag{9}$$

where $\boldsymbol{x}_{\text{mask}} \in \mathbb{R}^{L_{\text{m}}\times D}$ and $\boldsymbol{x}_{\text{vis}} \in \mathbb{R}^{L_{\text{v}}\times D}$ denote the masked and visible tokens, respectively, and MASK$(\cdot)$ is a task-dependent masking operator. Here, L_{m} and L_{v} represent the numbers of masked and visible tokens, respectively. For the denoising pretraining task, the input $\boldsymbol{x}$ is downsampled and interpolated before being fed into the network, with all tokens passed to the encoder without masking.

Encoder

The encoder first applies positional encoding to the preprocessed tokens and then processes them with a stack of transformer blocks. The positional encoding adopts the same space–time–frequency (STF) absolute positional encoding as WiFo, thereby embedding the three-dimensional coordinates of each token's associated patch in the original 3D CSI. This process can be formulated as $\boldsymbol{x}_{\text{enc}} = f_{\text{enc}}(\boldsymbol{x}_{\text{vis}} + \boldsymbol{P}_{\text{enc}})$, where $\boldsymbol{x}_{\text{enc}} \in \mathbb{R}^{L\times D}$ is the output of the encoder, $\boldsymbol{P}_{\text{enc}} \in \mathbb{R}^{L\times D}$ represents the positional encoding for the encoder, and f_{enc} denotes the encoder operation. To handle diverse CSI distributions and heterogeneous pretraining tasks, we propose a CSI-SMoE architecture that replaces the FFN in the vanilla transformer block. It comprises M expert networks $\{E_1, ..., E_M\}$ and four gating networks $\left\{G^1, G^2, G^3, G^4\right\}$, which respectively correspond to four pretraining tasks: random-masked reconstruction, time-masked reconstruction, frequency-masked reconstruction, and interpolation denoising. Each expert network is implemented with an identical FFN, while the gating network selectively activates K_{e} out of M experts and assigns weights according to the input token $\tilde{\boldsymbol{x}}$. For j-th pretraining tasks, the output of its gating network is derived as

$$G^j(\tilde{\boldsymbol{x}}) = \text{Top-K}(\text{softmax}(g(\tilde{\boldsymbol{x}})), K_{\text{e}}). \tag{10}$$

Here, $g(\cdot)$ denotes the learnable network within the gating network, which in practice is implemented as a fully-connected layer. The combination of the softmax function and the Top-K$(\cdot, K)$ operator remains K largest elements of the vector while setting the remaining entries to zero. The resultant output $\hat{\boldsymbol{x}}$ of the MoE layer is the weighted sum of the outputs from the K_{e} selected activated experts, derived as

$$\hat{\boldsymbol{x}} = \sum_{i=1}^{M} G^j(\tilde{\boldsymbol{x}})_i E_i(\tilde{\boldsymbol{x}}), \tag{11}$$

where $G^j(\tilde{\boldsymbol{x}})_i$ denotes the activation weight of the i-th expert for the j-th pretraining task, and $E_i(\tilde{\boldsymbol{x}})$ denotes the output of the i-th expert.

Decoder

In the masked reconstruction task, the encoder output is concatenated with learnable mask tokens. In contrast, the interpolation denoising task proceeds without them. Subsequently, the decoder applies STF positional encoding to all input tokens and processes them through multiple transformer blocks, which can be formulated as $\boldsymbol{x}_{\text{dec}} = f_{\text{dec}}(\boldsymbol{x}_{\text{dec}-\text{in}} + \boldsymbol{P}_{\text{dec}})$, where $\boldsymbol{x}_{\text{dec}-\text{in}} \in \mathbb{R}^{L\times D}$ and $\boldsymbol{x}_{\text{dec}} \in \mathbb{R}^{L\times D}$ denote the decoder input and output, respectively. $\boldsymbol{P}_{\text{dec}} \in \mathbb{R}^{L\times D}$ denotes the positional encoding, and f_{dec} denotes the decoder mapping. The decoder uses the same CSI-SMoE architecture as the encoder, but is lighter. Finally, the decoder maps $\boldsymbol{x}_{\text{dec}}$ to the complete reconstructed CSI via a linear projection and dimensional reshaping.

5 Supplementary Note 5. Details of pretraining scheme and applications

Phase 1: Mixed masking and denoising pretraining

We first pretrain WiFo-2 using a hybrid self-supervised framework that simultaneously learns expressive CSI representations and boosts performance on downstream channel prediction and estimation. The pretraining suite comprises four tasks: random-masked reconstruction, time-masked reconstruction, frequency-masked reconstruction, and interpolation denoising. On the coarse-grained CSI dataset, we pretrain WiFo-2 with three masked-reconstruction objectives to build generic masked-modeling capacity and improve performance on time and frequency domain prediction. On the fine-grained dataset, we apply the interpolation denoising pretraining scheme to enhance CSI denoising performance specifically for channel estimation.

Random-masked reconstruction. At a high masking ratio r_{R}, tokens are randomly omitted. This objective requires the network to reconstruct the full CSI from incompletely observed inputs, strengthening its modeling of the joint space–time–frequency structure.

Time-masked reconstruction. Along the time axis, a proportion r_{T} of tokens is masked, requiring the model to infer the final r_{T} portion of CSI in the temporal dimension from the observed $1 - r_{\mathrm{T}}$ portion. This objective trains the network to infer future CSI dynamics from historical observations, improving time-domain channel prediction.

Frequency-masked reconstruction. Tokens with frequency indices above a specified threshold are masked at a ratio r_{F}. This objective compels the network to reconstruct CSI from partially observed spectra and strengthens learning of inter-band dependencies across neighboring subbands, thereby improving performance on frequency domain channel prediction.

Interpolation denoising. We preprocess the raw 3D CSI by retaining CSI points at a subset of space-time-frequency locations to emulate partial pilot placement. A coarse complete CSI is then produced by linear interpolation and passed to the network without masking. In this regime, WiFo-2 functions as a denoiser, yielding a refined CSI estimate and enhancing channel estimation.

During pretraining, the loss function combines a reconstruction loss $\mathcal{L}_{\mathrm{rec}}$ and an auxiliary load-balancing loss $\mathcal{L}_{\mathrm{load}}$. Denote the reconstructed CSI on the target subset ω and its corresponding ground truth by $\boldsymbol{H}_{\omega}^{\mathrm{rec}}$ and bmH_{ω}, respectively. The MSE is adopted as the reconstruction loss, i.e.,

$$\mathcal{L}_{\mathrm{rec}} = \|\boldsymbol{H}_{\omega} - \boldsymbol{H}_{\omega}^{\mathrm{rec}}\|_F^2. \tag{12}$$

In masked-reconstruction tasks, ω comprises the masked positions. In the interpolation denoising task, ω is the entire set. In addition, to mitigate load imbalance induced by the sparse MoE, we add a load-balancing loss. For task j at an MoE layer, given a

batch $\mathcal{B}$ with T_{B} tokens, the load-balancing loss is given by

$$\mathcal{L}_{\text{load}} = M \sum_{i=1}^{M} \mathcal{D}_i P_i. \tag{13}$$

Here, $\mathcal{D}_i = \frac{1}{K_{\mathrm{e}} T_{\mathrm{B}}} \sum_{x \in \mathcal{B}} \mathbf{1}\left\{ \arg\max_k G^j(\tilde{\boldsymbol{x}})_k = i \right\}$ denotes the fraction of tokens routed to expert i across the batch, $P_i = \frac{1}{T_{\mathrm{B}}} \sum_{x \in \mathcal{B}} G^j(\tilde{\boldsymbol{x}})_i$ denotes the average activation weight assigned to expert i by the j-th gating network.

Phase 2: Confidence-enhanced pretraining

The zero-shot generalization performance of a model largely depends on the degree of distributional mismatch between the test scenario and the pretraining dataset. Despite extensive pretraining on a large-scale dataset in the first stage, certain corner cases can cause performance degradation, which is unacceptable for reliability-oriented communication systems. To address this, a confidence-enhanced pretraining scheme is designed to enable the pretrained WiFo-2 to actively assess and quantify its inference performance. This approach ensures more robust zero-shot generalization and offers precise channel quality priors for subsequent communication processes.

As shown in Fig. S2, a learnable channel-quality indicator token is added at the beginning of the input token sequence for the WiFo-2 decoder. During the decoder's forward inference, this token leverages the self-attention mechanism to perceive both the feature distribution of the input data and the proportion of masked tokens, thereby endowing the model with inference-awareness capability. To maintain the robust channel representations acquired during the first phase pretraining of WiFo-2, without modifying its original output for channel prediction and estimation, we introduce a prefix-isolated attention mask. In this design, the original tokens attend exclusively to one another, effectively isolating them from the influence of the confidence token. An additional MLP-based output head is introduced to predict the NMSE of channel reconstruction, with the MSE adopted as the loss function. Notably, this pretraining scheme allows an easy decoupling between the confidence output capability and different channel reconstruction tasks. Therefore, we train a separate learnable token and confidence output head for each task to avoid the common objective conflicts in multi-task training, thereby improving the accuracy and stability of the confidence outputs.

Applications

After two-stage pretraining, WiFo-2 can be directly applied to CSI reconstruction tasks. For channel prediction tasks, the target CSI segment is zero-padded along the temporal or frequency axis, and the corresponding masking type is applied. The task-aware gating network is selected for expert activation, and the predicted CSI is then obtained from the corresponding portion of the reconstructed CSI. For channel

estimation, the coarsely estimated CSI is fed into the network, and the interpolation denoising gating network is activated. In addition to the reconstruction result, a confidence output is also produced.

Moreover, WiFo-2 can be efficiently applied to diverse downstream tasks via few-shot fine-tuning. Specifically, leveraging the encoder's feature extraction ability and the decoder's CSI reconstruction ability, we design fine-tuning architectures for both uni-modal and multi-modal tasks.

Fine-tuning for uni-modal tasks. The encoder-only design is adopted for CSI feature extraction, wherein the WiFo-2 encoder is frozen and a trainable classification or regression output head is attached to enable scenario classification, beam prediction, user localization, AoA estimation, and Doppler estimation. The encoder–decoder architecture is employed for tasks whose outputs are CSI, leveraging WiFo-2's intrinsic CSI reconstruction capability to perform cross-band channel prediction and CSI feedback directly.

Fine-tuning for multi-modal tasks. The encoder-only architecture is employed for signal detection, where a frozen WiFo-2 encoder and a trainable signal encoder process the CSI and received signal, respectively, followed by a fusion encoder and detection head to output the detected symbol. The encoder-only architecture is applied for vision-aided channel prediction, with a frozen image encoder and a trainable connector converting images into tokens that are concatenated to the WiFo-2 decoder's input token sequence.

During fine-tuning, we apply a task-specific gating scheme. Since the added gating network imposes only minor adaptation overhead, this design enables efficient transfer of general CSI knowledge across various tasks.

6 Supplementary Note 6. Details of network and pretraining experiment settings

Table S6 The network configuration of WiFo-2 variants. The "enc." and "dec." denote the encoder and decoder, respectively.

Version	Depth (enc. /dec.)	Heads	Experts (active/total)	Expert dimension	Params (active/total)
Tiny	6/4	4	2/8	64	0.3M/0.6M
Little	6/4	4	2/8	128	1.1M/2.2M
Small	6/4	8	2/8	256	4.2M/8.3M
Base	6/4	8	2/8	512	16.5M/32.4M
Large	8/6	12	2/8	768	50.9M/100.9M

WiFo-2 comprises 5 versions, including tiny, little, small, base, and large, and detailed parameters for each are listed in Table S6. For each version of WiFo-2, 2 experts are selectively activated out of the 8. We use a patch size of 4 along the antenna, time, and subcarrier dimensions. During the two-phase pretraining, we add complex Gaussian noise to the input CSI, with SNR randomly set to 10–25dB. For

the time- and frequency-masked reconstruction pretraining tasks, the mask ratio is randomly sampled from 10%–50%. For the random-masked reconstruction pretraining tasks, the mask ratio is fixed as 85%. For the interpolation–denoising task, pilot-symbol placement proportions over (time, antenna, subcarrier) are randomly chosen in the range (1/8, 1, 1/24) to (1/4, 1, 1/6). All models are trained on 8 NVIDIA GeForce RTX 5090-32G GPUs with BF16 precision.

For first-phase pretraining, we trained for 250 epochs with a batch size of 128. We used AdamW ($\beta_1 = 0.9$, $\beta_2 = 0.999$) with a weight decay of 0.05. The load-balancing loss is weighted by 0.03. We employed a cosine-decay learning-rate schedule with 5 warmup epochs. For the base and small versions, the base learning rate is 5×10^{-4} and the minimum learning rate is 3×10^{-4}. For the large version, the base learning rate is 3×10^{-4} and the minimum learning rate is 6×10^{-5}.

For second-stage pretraining, we trained for 50 epochs with a batch size of 128, again using AdamW ($\beta_1 = 0.9$, $\beta_2 = 0.999$) with a weight decay of 0.001 and a cosine-decay schedule with 5 warmup epochs. Across all model sizes, the base learning rate was 1×10^{-4} with a minimum of 1×10^{-5}.

7 Supplementary Note 7. Dataset, baselines, and implementation details for evaluation

In this section, we provide a detailed account of the dataset construction, the implementation details, and the baseline settings for each task.

Channel reconstruction. We evaluate the zero-shot generalization performance of WiFo-2 on the zero-shot split of the LH-CSI dataset. For each channel reconstruction task, we evaluate the zero-shot performance at both the 10 dB and 20 dB SNR, and under both the high and low prediction/estimation ratios. Details of the adopted baselines are presented below.

(1) **Transformer**[19]: The transformer-based predictor in [19] is proposed for parallel channel prediction. For a fair comparison, it uses the same CSI embedding pipeline as WiFo-2. Specifically, the raw 3D CSI is first partitioned into patches and embedded into tokens, which are then processed by the network. The token dimension is set to 128, and the encoder and decoder depths are 5 and 8, respectively. In our experiments, the model is trained and evaluated for both time-domain and frequency-domain CSI prediction.

(2) **3D ResNet**[20]: A 3D ResNet (SlowFast-style) originally devised for video recognition [20] leverages spatiotemporal convolutions to model volumetric dependencies. In our configuration, the 50-layer network ingests CSI tensors to capture three-dimensional correlations across time, frequency, and antenna dimensions, and is trained to perform both time-domain and frequency-domain channel prediction.

(3) **LSTM**[21]: Long short-term memory (LSTM) is proposed for sequential processing to overcome the vanishing gradient problem. For time-domain channel prediction, the antenna and frequency dimensions are flattened and fed as features to the LSTM. For frequency-domain prediction, we analogously flatten the time and antenna dimensions.

(4) **LLM-based scheme**[22]: LLM4CP fine-tunes GPT-2 for cross-modal knowledge transfer in channel prediction [22]. We equip LLM4CP with the same 3D patching module as WiFo-2 so that it can handle 3D CSI. By analogy, we also evaluate a fine-tuned LLM for channel estimation.

(5) **Interpolation**: We include interpolation baselines for both prediction and estimation. For channel prediction, future CSI is obtained by first-order linear extrapolation from historical observations. For channel estimation, we use bilinear interpolation to reconstruct missing CSI entries.

(6) **PAD**[23]: PAD is a Prony-based angular-delay domain channel prediction method that is based on the angle-delay-doppler structure of the channel. We use PAD only for time-domain prediction and set the predictor order to $N = 4$.

(7) **Time-MoE**[24]: Time-MoE is a large-scale time-series foundation model with strong zero-shot forecasting ability [24]. We evaluate the Time-MoE-200M checkpoint strictly under a zero-shot protocol, using the publicly released pretrained model without fine-tuning, prompt tuning, or in-domain adaptation. By parallelizing the remaining two dimensions, we transform time-domain and frequency-domain channel prediction into a one-dimensional sequence prediction problem, which can be readily handled by Time-MoE.

(8) **ChannelNet**[25]: ChannelNet formulates channel estimation using image super-resolution and restoration techniques within a CNN framework. Following [25], we employ a 2-layer CNN for super-resolution and a 20-layer CNN for restoration. To handle 3D CSI, the antenna dimension is processed in parallel across channels.

(9) **Channelformer**[26]: Channelformer adopts an encoder–decoder architecture tailored to channel estimation. In line with the configuration in [26], we use 5 encoder layers and 12 decoder layers, and parallelize over the antenna dimension to accommodate 3D CSI.

For all task-specific AI models and LLM-based schemes, the SNR during training is randomly chosen in the range 10–25 dB, identical to WiFo-2 for a fair comparison. We use the AdamW optimizer with $\beta_1 = 0.9$, $\beta_2 = 0.999$ and a weight decay of 0.001. For channel prediction tasks, all task-specific and LLM-based schemes are trained for 50 epochs with a fixed learning rate of 4×10^{-4} and a batch size of 64. For channel estimation tasks, the learning rate is adjusted to 1×10^{-4}, with other settings unchanged.

Table S7 The fine-tuning setting of WiFo-2 across scenario classification, sub-6 GHz to mmWave beam prediction, and wireless localization.

**Task**	Scenario classification	Sub-6 GHz to mmWave beam prediction	Wireless localization
**Optimizer**	AdamW	AdamW	AdamW
**Learning rate**	2×10^{-4}	1×10^{-4}	2×10^{-5}
**Weight decay**	1×10^{-4}	1×10^{-4}	1×10^{-4}
**Optimizer momentum**	(0.9, 0.999)	(0.9, 0.999)	(0.9, 0.999)
**Batch size**	256	256	256
**Max epoch**	100	100	200
**Loss function**	CrossEntropy	CrossEntropy	MSE

Table S8 The fine-tuning setting of WiFo-2 across Doppler estimation and vision-aided frequency-domain channel prediction.

**Task**	Doppler estimation	Vision-aided frequency-domain channel prediction
**Optimizer**	AdamW	AdamW
**Learning rate**	2×10^{-5}	1×10^{-4}
**Weight decay**	1×10^{-4}	1×10^{-4}
**Optimizer momentum**	(0.9, 0.999)	(0.9, 0.999)
**Batch size**	256	256
**Max epoch**	200	100
**Loss function**	MSE	MSE

Table S9 The fine-tuning setting of WiFo-2 across CSI feedback, AoA estimation, cross-band channel prediction, and signal detection.

**Task**	CSI feedback	AoA estimation	Cross-band channel prediction	Signal detection
**Optimizer**	AdamW	AdamW	AdamW	AdamW
**Learning rate**	6×10^{-5}	2×10^{-5}	4×10^{-5}	2×10^{-5}
**Weight decay**	1×10^{-4}	1×10^{-4}	1×10^{-4}	1×10^{-4}
**Optimizer momentum**	(0.9, 0.999)	(0.9, 0.999)	(0.9, 0.999)	(0.9, 0.999)
**Batch size**	256	256	256	256
**Max epoch**	300	200	100	100
**Loss function**	MSE	MSE	MSE	CrossEntropy

Scenario classification. We used QuaDRiGa [12] to synthesize a scenario-classification dataset with 1,000 UMi-LoS and 1,000 UMi-NLoS CSI samples at a center frequency of 2.5 GHz and a bandwidth of 10 MHz. Each CSI sample is annotated with a scenario label and is organized along three axes, temporal, frequency, and spatial, with 24 consecutive time samples, 128 OFDM subcarriers, and measurements over a 2×4 UPA. The temporal sampling interval is 1 ms, and the subcarrier spacing is 90 kHz. The dataset is partitioned into training, validation, and test sets containing 1,000, 500, and 500 samples, respectively. Each wireless foundation model serves as an encoder for feature extraction, upon which we attach the same linear classification head to produce scenario labels. As for task-specific AI models, we adopt ST-CNN [27], a 3D CNN tailored to capture the joint temporal–frequency–spatial structure of CSI. The F1 score was used to evaluate the classification accuracy. Further training details are provided in Table S7.

Sub-6 GHz to mmWave beam prediction. This dataset was also generated using the QuaDRiGa [12] simulator under the UMi-LoS scenario. The dataset comprises 5,000 training samples, 1,000 validation samples, and 1,000 test samples, each containing paired sub-6 GHz CSI and mmWave CSI acquired at the same transmitter–receiver location. For the sub-6 GHz link, the carrier frequency is set to 2.5 GHz with a bandwidth of 10 MHz, employing a 1×16 ULA antenna configuration. For the mmWave link, the carrier frequency is 28 GHz with a bandwidth of 0.5 GHz, using a 1×64 ULA antenna configuration. Both the sub-6 GHz link and the mmWave link comprise 64 subcarriers. The mmWave system uses a 64-entry DFT codebook, and the optimal beam is the codeword that maximizes the average magnitude across all subcarriers. Each wireless foundation model serves as an encoder to extract features from

the sub-6 GHz channel, and the same linear layer is subsequently applied to predict the corresponding mmWave beam. For task-specific AI models, we adopt the same neural network architecture proposed in [28], which consists of five fully-connected layers with ReLU activation functions. Both the top-1 classification accuracy and SE are adopted as evaluation metrics. Specifically, the SE is computed based on the true millimeter-wave channel $\boldsymbol{h} \in \mathbb{C}^{N_t \times K}$ and the beamforming vector $\boldsymbol{w}_b \in \mathbb{C}^{N_t}$ from the DFT codebook corresponding to the beam index b predicted by each model, where N_t and K denote the number of antennas and subcarriers, respectively. The SE can be expressed as

$$R = \sum_{k=1}^{K} \log_2 \left(1 + \frac{|\boldsymbol{h}_k^{\mathrm{H}} \boldsymbol{w}_b|^2}{\sigma_n^2} \right), \tag{14}$$

where $\boldsymbol{h}_k^{\mathrm{H}}$ is kth column of $\boldsymbol{h}$. More training details are provided in Table S7.

Wireless Localization. We evaluate wireless localization on the dichasus-005x scenario [15] using 10,500 sample pairs consisting of CSI and user 2D positions. The system operates at the center frequency of 1.272 GHz with a bandwidth of 50 MHz. Each CSI sample spans 64 OFDM subcarriers in frequency and a 4×8 UPA in space. The dataset is divided into 10,000, 300, and 200 samples for training, validation, and testing. Wireless foundation models serve as an encoder for feature extraction. For the task-specific AI models, we adopt WiT [29], which utilizes multi-head self-attention to capture long-range dependencies across subcarriers and antenna elements jointly, learns discriminative representations from raw CSI, and regresses positions via a lightweight head. Root-mean-square error (RMSE) is adopted as the metric of the localization accuracy, and training details are provided in Table S7.

CSI Feedback. For the CSI feedback task, we constructed both the statistical channel modeling-based dataset and the real-world measurement-based dataset for validation. For the first dataset, we utilize QuaDRiGa [12] to generate the dataset under an Indoor LoS scenario. Each CSI sample corresponds to a carrier frequency of 3.5 GHz and a 1 MHz bandwidth, including 32 subcarriers and 32 antennas. For the second dataset, we derived it by processing the subset 40 of Argos [16], which corresponds to an outdoor scenario. Each CSI sample corresponds to a carrier frequency of 2.4 GHz and a 20 MHz bandwidth, including 32 subcarriers and 32 antennas. Both datasets contain 1,200 antenna-frequency 2D samples, which are split into 1,000 for training, 100 for validation, and 100 for testing. We adopt the same experimental setting as CsiNet [30]. The transmitter compresses the CSI to 25% and sends it, and the receiver reconstructs the original CSI. Because CSI compression and feedback typically use a two-network design, with an encoder deployed at the transmitter and a decoder deployed at the receiver, the wireless foundation model is employed on the receiver side to denoise the reconstructed CSI. Specifically, we first train CsiNet [30] on the constructed dataset, and then the wireless foundation model further refines the CSI reconstructed by CsiNet to improve reconstruction accuracy. During this process, only the input embedding, output head, and the first transformer block are updated, while most parameters remain frozen. As a task-specific baseline, we adopt the TransNet [31] scheme, which represents the SOTA in compact CSI feedback networks. Similarly, we

employ both NMSE and SE as performance metrics, where SE is computed exactly as for the vision-aided frequency-domain channel prediction task. More training details are given in Table S9.

AoA Estimation. For the AoA estimation task, we select the I1_2p4 scenario of DeepMIMO [17] to generate 1,200 samples, where 1,000, 100, and 100 samples are utilized for training, validation, and testing, respectively. Each sample contains a CSI and a corresponding AoA of its dominant path. The center frequency is 2.5 GHz, and the BS is equipped with a ULA with 32 antennas. Each CSI sample spans a 40 MHz bandwidth with 64 subcarriers. Each wireless foundation model acts as a CSI feature-extraction encoder, followed by a linear layer that outputs the AoA estimate. For the task-specific AI baseline, we build a ResNet-based network similar to [4] for AoA estimation. We adopt the mean absolute error (MAE) between the predicted AoA and the ground truth as the evaluation metric. Details of the training configuration are provided in Table S9.

Doppler Estimation. For the Doppler estimation task, we utilize QuaDRiGa [12] to generate an LEO satellite channel dataset under the NTN_Rural_LOS scenario, where the satellite altitude is set to 600 km. Each CSI sample corresponds to a carrier frequency of 2 GHz. Both the satellite and the user terminal are equipped with a single antenna. Each sample is represented as a time-frequency 2D CSI, where the time-domain dimension contains 64 sampling points with a sampling interval of 5 μs, and the frequency-domain dimension contains 32 subcarriers over a 30 MHz bandwidth. The dataset contains 1,800 samples in total, which are split into 1,000 for training, 300 for validation, and 500 for testing. Each sample is associated with the corresponding Doppler shift as the ground-truth label. Each wireless foundation model acts as a CSI feature-extraction encoder, followed by a linear layer that outputs the Doppler estimate. For the task-specific AI baseline, we adopt a CNN-Transformer style solution [32]. We adopt the MAE between the predicted Doppler shift and the ground truth as the evaluation metric. Details of the training configuration are provided in Table S8.

Cross-band channel prediction. We construct two datasets for evaluation. The first dataset for cross-band channel prediction is generated using QuaDRiGa [12] under the RMa-LoS scenario at 2.5 GHz center frequency. Each sample contains paired 3D CSI from two bands whose center frequencies are 3 MHz apart, where both CSI have 4 time samples and 8 antennas. Another dataset is generated according to the dichasus-ca0x dataset with a center frequency of 1.27GHz. The measured CSI is collected in an industrial environment, specifically in a LoS area within the ARENA2036 research factory campus. Each sample contains paired 3D CSI from two bands whose center frequencies are 12.5 MHz apart, where both CSI have 4 time samples and 32 antennas. Both datasets consist of 2,200 samples, partitioned into 2,000 for training, 100 for validation, and 100 for testing. The task aims to infer the latter band's center-subcarrier CSI from 32 subcarriers of the former band. In this task, each wireless foundation model primarily serves as a feature extractor. The extracted channel representations are linearly mapped to the corresponding target-band channels through a fully connected layer. As a task-specific baseline, we employ RF-Diffusion [29], a powerful diffusion-based generative model for RF data synthesis. It demonstrates strong performance in cross-band channel prediction by effectively capturing temporal–frequency

domain dependencies through diffusion processes. NMSE and SE are adopted as evaluation metrics to assess the performance of these models, with SE computed in the same way as in the CSI feedback task. More training details are given in Table S9.

Vision-aided Frequency-domain Channel Prediction. In the urban crossroad scenario of the SynthSoM [33] dataset, we perform vision-aided frequency-domain channel prediction. The dataset comprises 120 samples, each containing spatially aligned 2D CSI data and RGB images with a resolution of 720×1280. The channel data is measured at 28 GHz with a 10 MHz bandwidth, including 64 subcarriers and 16 antennas. The dataset is partitioned into training, validation, and test subsets containing 100, 10, and 10 samples, respectively. For WiFo-2, we employ a pretrained ResNet-18 [34] to extract visual features, which are linearly projected and concatenated to the decoder's input token sequence. During fine-tuning, the RGB feature projection layer, the first layer and output head of the decoder, and the newly introduced task-specific gate in the encoder are activated, while all other parameters remain frozen. For LWM and SWIT, which lack a channel reconstruction decoder, we concatenate the extracted channel and RGB features and use a linear layer to reconstruct the predicted channel. As a task-specific baseline, we adopt a vision and CSI fusion architecture similar to [35], and the CSI encoder extracts features with the Swin Transformer architecture. We adopt the NMSE, which measures the reconstruction accuracy, and the SE, which directly reflects the capacity of the communication system, as the evaluation metrics. Specifically, we compute the matched-filter precoder $\boldsymbol{P} \in \mathbb{C}^{N \times K}$ from the predicted channel $\hat{\boldsymbol{h}} \in \mathbb{C}^{N \times K}$. Assuming the true channel is $\bar{\boldsymbol{h}} \in \mathbb{C}^{N \times K}$, the resulting SE can be computed as

$$R = \sum_{k=1}^{K} \log_2 \left(1 + \frac{|\bar{\boldsymbol{h}}_k^{\mathrm{H}} \boldsymbol{P}_k|^2}{\sigma_n^2} \right), \tag{15}$$

where $\bar{\boldsymbol{h}}_k^{\mathrm{H}}$ and $\boldsymbol{P}_k$ denote the k-th column of $\bar{\boldsymbol{h}}$ and $\boldsymbol{P}$, respectively. More training details are provided in Table S8.

Signal detection. The QuaDRiGa [12] simulator is employed to generate the dataset for signal detection under the UMi-NLoS scenario at 2.5 GHz. A MISO system with single-carrier transmission and quadrature phase shift keying (QPSK)-modulated data symbols is considered. The receiver is equipped with 16 antennas, and the dataset comprises 24,000 samples, partitioned into 20,000 for training, 1,000 for validation, and 3,000 for testing. Each sample contains the received signal, the estimated channel matrix, and the transmitted symbols. To emulate practical system interference, complex Gaussian white noise with an SNR of 10 dB is added to the channel matrix. Wireless foundation models are utilized to extract CSI features, while a parallel DNN branch extracts features from the received signal. The two feature vectors are concatenated to form a fused representation, which is then passed through a linear output head to produce the detection results. During fine-tuning, the encoder that processes the received signal, the linear output head, and the newly introduced task-specific gate in the WiFo-2 encoder are activated, while all other parameters remain frozen. As a task-specific AI baseline, we adopt OAMPNet [36], a model-driven signal detection

network that achieves efficient training and strong performance with minimal parameter overhead, particularly under limited data scenarios. We use the symbol error rate (SER) to quantify the detection accuracy, defined as the ratio of incorrectly detected symbols to the total number of transmitted symbols. More training details are shown in Table S9.

8 Supplementary Note 8. Results on in-distribution channel reconstruction performance

Table S10 presents the in-distribution channel reconstruction performance of WiFo-2 and its comparison with the baselines.

Table S10 In-distribution channel reconstruction performance of WiFo-2 and other baselines.

Configurations	Task	Transformer	3D ResNet	LSTM	ChannelNet	Channelformer	LLM-based	Interpolation	PAD	Time-MoE	WiFo-2
SNR=20dB &low ratio	CP-F	-6.514	-7.792	-4.985	-	-	-9.429	1.232	-	-4.907	**-13.321**
	CP-T	-7.534	-11.253	-5.423	-	-	-11.445	0.793	-5.619	-5.560	**-15.384**
	CE	-	-	-	-16.229	-15.098	-15.016	-3.683	-	-	**-18.629**
SNR=20dB &high ratio	CP-F	-4.962	-6.239	-3.852	-	-	-7.582	3.636	-	-2.381	**-10.639**
	CP-T	-5.575	-7.662	-4.028	-	-	-8.404	4.313	5.790	-2.999	**-11.077**
	CE	-	-	-	-12.514	-10.550	-12.479	-1.162	-	-	**-14.190**
SNR=10dB &low ratio	CP-F	-6.177	-6.741	-4.954	-	-	-9.059	1.297	-	-3.384	**-12.583**
	CP-T	-7.227	-8.987	-5.415	-	-	-10.491	0.960	-3.138	-3.816	**-14.278**
	CE	-	-	-	-14.816	-15.097	-14.839	-2.185	-	-	**-16.873**
SNR=10dB &high ratio	CP-F	-4.697	-5.407	-3.840	-	-	-7.331	3.746	-	-1.865	**-10.016**
	CP-T	-5.404	-6.309	-4.026	-	-	-7.923	4.550	-1.625	-2.327	**-10.322**
	CE	-	-	-	-11.778	-10.552	-12.427	-0.635	-	-	**-12.747**

The table reports NMSE values across all pretraining datasets in dB. CP-T and CP-F refer to the time-domain and frequency-domain channel prediction tasks, respectively, and CE refers to the channel estimation task. Values in **bold** indicate the best performance in each row.

9 Supplementary Note 9. Additional results for zero-shot channel reconstruction

Zero-shot channel reconstruction performance of WiFo-2 across varying SNRs and reconstruction ratios

The zero-shot channel reconstruction results of WiFo-2 and the baselines across different SNRs and reconstruction ratios are reported in Tables S11, S12, S13, and S14.

Table S11 Zero-shot channel reconstruction performance of WiFo-2 compared to other baselines across various generalization sub-datasets. A low prediction and estimation ratio and a 20 dB SNR are used.

		Full-shot						Zero-shot			
Dataset	Task	Transformer	3D ResNet	LSTM	ChannelNet	Channelformer	LLM-based	Interpolation	PAD	Time-MoE	WiFo-2
QC17	CP-T	-4.046	-9.782	-3.971	-	-	-8.631	4.911	-2.929	-4.294	**-13.559**
	CP-F	-1.274	-3.611	-2.164	-	-	-5.265	2.556	-	-0.855	**-11.813**
QC18	CP-T	-6.854	-10.737	-5.692	-	-	-10.775	3.977	-5.632	-4.610	**-15.130**
	CP-F	-8.717	-9.135	-4.989	-	-	-9.112	0.369	-	-4.903	**-15.233**
QC19	CP-T	-2.358	-6.588	-2.024	-	-	-6.075	3.050	-2.093	-1.502	**-10.586**
	CP-F	-0.078	-4.550	-4.735	-	-	-5.464	-0.230	-	-4.963	**-13.710**
AC6	CP-F	-1.173	-12.669	-13.206	-	-	**-14.032**	4.900	-	-2.948	-12.282
AC7	CP-F	-3.432	-9.847	-7.287	-	-	**-10.242**	4.584	-	-4.479	-9.216
RMC8	CP-T	-17.419	-21.152	-18.835	-	-	**-21.173**	-18.504	-10.538	-17.815	-20.944
	CP-F	-11.929	-14.074	-12.540	-	-	**-16.099**	0.048	-	-11.136	-14.096
RMC9	CP-T	-13.931	-15.894	-14.957	-	-	-15.829	-13.788	-6.438	-6.479	**-15.919**
	CP-F	-11.410	-11.938	-9.515	-	-	**-12.605**	0.059	-	1.128	-11.917
DEC3	CP-T	-5.644	-9.439	-7.403	-	-	-9.060	2.608	-6.203	-1.768	**-9.951**
	CP-F	-5.929	**-17.052**	-8.745	-	-	-15.270	-5.743	-	-11.113	-15.170
RTC3	CP-T	-16.402	-22.736	-20.880	-	-	-18.242	-15.261	**-23.873**	-9.029	-21.660
	CP-F	-17.233	-21.160	-19.335	-	-	-18.738	-11.939	-	-7.259	**-22.974**
QF17	CE	-	-	-	-11.420	-6.644	-10.898	-8.037	-	-	**-14.525**
QF18	CE	-	-	-	-19.333	-6.075	-17.783	-15.315	-	-	**-24.468**
QF19	CE	-	-	-	-11.792	-5.213	-10.482	-8.918	-	-	**-14.010**
RMF8	CE	-	-	-	-17.640	-16.365	-18.048	1.647	-	-	**-20.588**
RMF9	CE	-	-	-	-14.456	-13.273	-15.368	1.702	-	-	**-15.994**
DEF3	CE	-	-	-	-19.520	-13.237	-18.775	-15.633	-	-	**-21.515**
Average	CP-F	-5.003	-8.764	-6.431	-	-	-9.715	1.306	-	-3.559	**-12.953**
	CP-T	-6.589	-10.958	-6.596	-	-	-10.386	1.337	-5.529	-4.546	**-13.626**
	CE	-	-	-	-14.473	-8.326	-13.882	-2.619	-	-	**-17.007**

The table reports NMSE values in dB. CP-T and CP-F refer to the time-domain and frequency-domain channel prediction tasks, respectively, and CE refers to the channel estimation task. Values in **bold** indicate the best performance in each row.

Cross-source generalization across dataset sources

WiFo-2 incorporates eight data sources, each obtained using different simulation methods or acquisition devices. To evaluate the cross-source generalization capability of WiFo-2, we pretrained the large version of WiFo-2 on the LH-CSI datasets, excluding the PKU-RM source (i.e., RMC1-RMC7 and RMF1-RMF7), comprising 14 datasets in total, and then evaluated its generalization performance on PKU-RM. Specifically, we tested its zero-shot channel prediction and estimation performance under an SNR of 20 dB and low reconstruction ratios, as shown in Tables S15 and S16.

For the channel prediction task, excluding PKU-RM from pretraining leads to a performance degradation of approximately 2.329 dB compared with pretraining on the complete LH-CSI dataset. This degradation can be attributed to the transfer loss

Table S12 Zero-shot channel reconstruction performance of WiFo-2 compared to other baselines across various generalization sub-datasets. A low prediction and estimation ratio and a 10 dB SNR are used.

		Full-shot						Zero-shot			
Dataset	Task	Transformer	3D ResNet	LSTM	ChannelNet	Channelformer	LLM-based	Interpolation	PAD	Time-MoE	WiFo-2
QC17	CP-T	-3.626	-6.819	-3.954	-	-	-7.024	4.986	-0.707	-1.315	**-11.825**
	CP-F	-1.183	-3.114	-2.136	-	-	-4.997	2.586	-	0.019	**-10.580**
QC18	CP-T	-6.410	-7.978	-5.705	-	-	-9.289	4.064	-3.915	-3.012	**-13.492**
	CP-F	-7.870	-7.545	-4.965	-	-	-8.428	0.420	-	-4.701	**-13.320**
QC19	CP-T	-2.225	-5.038	-2.024	-	-	-5.521	3.163	-0.319	-0.078	**-9.887**
	CP-F	-4.508	-4.016	-2.391	-	-	-5.387	-0.102	-	-6.666	**-12.924**
AC6	CP-F	-1.070	-7.201	-13.145	-	-	**-13.296**	4.929	-	-1.978	-11.281
AC7	CP-F	-3.139	-7.981	-7.278	-	-	**-9.856**	4.627	-	-3.973	-8.685
RMC8	CP-T	-16.775	-18.719	-18.636	-	-	**-20.503**	-11.896	-4.305	-11.582	-20.295
	CP-F	-11.521	-12.902	-12.326	-	-	**-15.400**	0.115	-	-8.486	-13.438
RMC9	CP-T	-13.680	-15.221	-14.838	-	-	-15.519	-10.257	-3.665	-5.068	**-15.636**
	CP-F	-11.137	-11.337	-9.354	-	-	**-12.204**	0.127	-	1.785	-11.480
DEC3	CP-T	-5.505	-7.974	-7.407	-	-	-8.728	2.730	-5.163	-1.536	**-9.464**
	CP-F	-5.674	-14.050	-8.742	-	-	-14.055	-5.544	-	-8.279	**-14.359**
RTC3	CP-T	-16.261	-19.897	-20.872	-	-	-17.835	-11.051	-20.461	-6.086	**-20.924**
	CP-F	-17.128	-18.922	-19.281	-	-	-18.528	-11.155	-	-5.315	**-21.864**
QF17	CE	-	-	-	-11.420	-6.644	-10.898	-5.928	-	-	**-13.426**
QF18	CE	-	-	-	**-19.333**	-6.075	-17.783	-8.285	-	-	-18.001
QF19	CE	-	-	-	-11.792	-5.213	-10.482	-6.251	-	-	**-12.778**
RMF8	CE	-	-	-	-17.640	-16.365	-18.048	2.671	-	-	**-20.096**
RMF9	CE	-	-	-	-14.456	-13.273	-15.368	2.719	-	-	**-15.788**
DEF3	CE	-	-	-	**-18.634**	-13.224	-18.628	-8.761	-	-	-18.014
Average	CP-F	-4.795	-7.535	-6.398	-	-	-9.364	1.363	-	-2.834	**-12.104**
	CP-T	-6.327	-8.938	-6.590	-	-	-9.507	1.499	-3.266	-2.949	**-12.758**
	CE	-	-	-	-14.422	-8.325	-13.874	-1.255	-	-	**-15.581**

The table reports NMSE values in dB. CP-T and CP-F refer to the time-domain and frequency-domain channel prediction tasks, respectively, and CE refers to the channel estimation task. Values in **bold** indicate the best performance in each row.

caused by the distribution shift across data sources. Nevertheless, WiFo-2 still maintains acceptable zero-shot channel prediction performance, reaching a level comparable to that of task-specific models trained with 9,000 samples.

For the channel estimation task, excluding PKU-RM from pretraining results in a performance loss of approximately 2.386 dB compared with pretraining on the complete LH-CSI dataset. Despite this loss, WiFo-2 still achieves acceptable zero-shot channel estimation performance and even outperforms the full-shot performance of Channelformer.

These results demonstrate that, through pretraining on large-scale heterogeneous CSI data, WiFo-2 learns generalizable CSI representations and exhibits strong zero-shot generalization capability even on previously unseen data-collection sources.

Table S13 Zero-shot channel reconstruction performance of WiFo-2 compared to other baselines across various generalization sub-datasets. A high prediction and estimation ratio and a 20 dB SNR are used.

		Full-shot						Zero-shot			
Dataset	Task	Transformer	3D ResNet	LSTM	ChannelNet	Channelformer	LLM-based	Interpolation	PAD	Time-MoE	WiFo-2
QC17	CP-T	-2.508	-5.627	-1.683	-	-	-5.264	8.791	-0.340	-0.044	**-8.749**
	CP-F	-0.571	-2.175	-1.149	-	-	-3.088	5.596	-	0.782	**-7.405**
QC18	CP-T	-4.386	-6.299	-2.862	-	-	-7.301	7.777	-1.714	-2.182	**-9.656**
	CP-F	-0.100	-5.974	-2.610	-	-	-6.809	3.478	-	-1.979	**-10.223**
QC19	CP-T	-0.985	-3.414	-1.415	-	-	-3.576	6.347	-0.217	0.903	**-6.835**
	CP-F	-0.071	-3.429	-1.198	-	-	-4.228	2.587	-	-6.134	**-10.574**
AC6	CP-F	-0.670	-12.328	-12.019	-	-	**-12.592**	8.071	-	0.556	-7.862
AC7	CP-F	-0.865	-7.069	-5.380	-	-	**-7.957**	7.613	-	-1.716	-5.589
RMC8	CP-T	-16.267	-18.493	-10.582	-	-	-19.444	-14.788	-9.105	-13.921	**-19.636**
	CP-F	-10.443	-9.726	-9.179	-	-	**-14.002**	0.071	-	-4.104	-9.919
RMC9	CP-T	-12.988	-14.176	-6.100	-	-	**-15.009**	-10.954	-5.224	-4.246	-14.949
	CP-F	-9.282	-9.058	-2.367	-	-	**-11.128**	0.105	-	0.590	-8.469
DEC3	CP-T	-3.636	-5.398	-5.388	-	-	**-6.965**	5.656	-0.002	-2.302	-5.038
	CP-F	-5.547	-13.409	-6.841	-	-	**-13.913**	-2.865	-	-5.044	-11.100
RTC3	CP-T	-16.603	**-20.916**	-19.252	-	-	-18.242	-12.066	-20.145	-4.814	-19.016
	CP-F	-13.916	-19.563	-17.508	-	-	-16.360	-8.615	-	-5.675	**-19.801**
QF17	CE	-	-	-	-6.266	-2.178	-7.091	-2.416	-	-	**-8.689**
QF18	CE	-	-	-	-18.625	-2.741	-16.861	-15.666	-	-	**-21.840**
QF19	CE	-	-	-	-8.240	-2.253	-7.990	-5.059	-	-	**-10.121**
RMF8	CE	-	-	-	-14.367	-9.424	-17.307	3.312	-	-	**-18.940**
RMF9	CE	-	-	-	-11.609	-8.651	-14.836	3.314	-	-	**-15.104**
DEF3	CE	-	-	-	**-17.855**	-10.575	-17.199	-12.936	-	-	-16.209
Average	CP-F	-2.517	-6.859	-4.288	-	-	-7.860	3.989	-	-1.759	**-8.987**
	CP-T	-4.927	-7.269	-4.382	-	-	-7.726	4.919	-2.450	-2.259	**-9.325**
	CE	-	-	-	-10.605	-4.620	-11.294	-0.578	-	-	**-12.915**

The table reports NMSE values in dB. CP-T and CP-F refer to the time-domain and frequency-domain channel prediction tasks, respectively, and CE refers to the channel estimation task. Values in **bold** indicate the best performance in each row.

Prediction performance across different prediction horizons

Furthermore, to characterize how prediction accuracy varies with the prediction horizon, we report the corresponding results of both time-domain and frequency-domain channel prediction under the low prediction-ratio setting at an SNR of 10 dB. Let T and K denote the numbers of time samples and subcarriers, respectively, in a given dataset. Under the low prediction-ratio setting, the time-domain and frequency-domain prediction horizons are defined as $T_p = T/4$ and $K_p = K/4$, respectively. Unlike the results reported in Tables S11, S12, S13, and S14, which are averaged over the entire prediction horizon, we further evaluate the average prediction performance over the first 25%, 50%, 75%, and 100% of T_p and K_p, as summarized in Table S17. The results are averaged across all zero-shot generalization splits of the LH-CSI dataset. The NMSE increases progressively as the prediction horizon becomes

Table S14 Zero-shot channel reconstruction performance of WiFo-2 compared to other baselines across various generalization sub-datasets. A high prediction and estimation ratio and a 10 dB SNR are used.

		Full-shot						Zero-shot			
Dataset	Task	Transformer	3D ResNet	LSTM	ChannelNet	Channelformer	LLM-based	Interpolation	PAD	Time-MoE	WiFo-2
QC17	CP-T	-2.255	-4.027	-1.672	-	-	-4.543	8.887	0.326	0.584	**-7.664**
	CP-F	-0.518	-1.838	-1.136	-	-	-2.925	5.644	-	0.848	**-6.691**
QC18	CP-T	-4.110	-4.871	-2.857	-	-	-6.560	7.894	-1.286	-1.739	**-8.745**
	CP-F	-0.099	-4.924	-2.595	-	-	-6.238	3.555	-	-1.246	**-9.075**
QC19	CP-T	-0.956	-2.547	-1.422	-	-	-3.400	6.511	0.874	0.787	**-6.378**
	CP-F	-0.068	-3.094	-1.188	-	-	-4.155	2.683	-	-5.856	**-9.973**
AC6	CP-F	-0.621	-7.754	-12.009	-	-	**-12.104**	8.114	-	1.071	-7.119
AC7	CP-F	-0.816	-5.315	-5.370	-	-	**-7.830**	7.677	-	-1.444	-5.174
RMC8	CP-T	-15.594	-15.437	-10.550	-	-	-18.760	-7.058	-3.590	-8.965	**-18.778**
	CP-F	-9.744	-8.915	-9.143	-	-	**-12.576**	0.258	-	-3.099	-9.355
RMC9	CP-T	-12.767	-13.044	-6.035	-	-	**-14.666**	-6.084	-2.613	-3.478	-14.587
	CP-F	-9.226	-8.629	-2.329	-	-	**-10.351**	0.292	-	0.563	-8.069
DEC3	CP-T	-3.611	-4.594	-5.414	-	-	**-6.894**	5.846	-0.248	-1.784	-4.928
	CP-F	-5.359	-11.028	-6.790	-	-	**-13.070**	-2.531	-	-3.855	-10.500
RTC3	CP-T	-16.395	-18.654	**-19.185**	-	-	-17.915	-6.537	-17.409	-3.419	-18.300
	CP-F	-13.694	-17.284	-17.422	-	-	-16.122	-7.497	-	-4.612	**-18.539**
QF17	CE	-	-	-	-6.266	-2.178	-7.091	-1.644	-	-	**-8.034**
QF18	CE	-	-	-	**-18.625**	-2.741	-16.861	-9.476	-	-	-15.676
QF19	CE	-	-	-	-8.240	-2.253	-7.990	-3.736	-	-	**-9.168**
RMF8	CE	-	-	-	-14.367	-9.424	-17.307	3.519	-	-	**-17.780**
RMF9	CE	-	-	-	-11.609	-8.651	**-14.836**	3.519	-	-	-14.544
DEF3	CE	-	-	-	**-17.256**	-10.582	-17.075	-8.164	-	-	-10.649
Average	CP-F	-2.463	-5.940	-4.268	-	-	-7.557	4.073	-	-1.342	**-8.364**
	CP-T	-4.788	-6.104	-4.376	-	-	-7.321	5.144	-1.468	-1.724	**-8.816**
	CE	-	-	-	-10.585	-4.620	-11.289	-0.095	-	-	**-11.314**

The table reports NMSE values in dB. CP-T and CP-F refer to the time-domain and frequency-domain channel prediction tasks, respectively, and CE refers to the channel estimation task. Values in **bold** indicate the best performance in each row.

longer. This degradation arises because temporal and frequency correlations weaken with increasing prediction distance, making long-range prediction more challenging. Therefore, in practical deployments, the prediction horizon should be appropriately selected according to the required prediction accuracy.

Additional experiments of task-specific baselines

To validate the rationale behind our task-specific baseline configurations, we systematically evaluate the scaling behavior of task-specific AI models with respect to both model capacity and training dataset size. Focusing on the frequency-domain channel prediction task within the QC18 dataset, we select 3D ResNet, the top-performing architecture among the three candidate task-specific schemes, as our benchmark. By

Table S15 Zero-shot results for channel prediction using WiFo-2 pretrained on LH-CSI datasets without PKU-RM. A low prediction ratio and a 20 dB SNR are used.

		Full-shot				Zero-shot				
Dataset	Task	Transformer	3D ResNet	LSTM	LLM-based	Interpolation	PAD	Time-MoE	WiFo-2	WiFo-2 (w/o RM)
RMC8	CP-T	-17.419	-21.152	-18.835	**-21.173**	-18.504	-10.538	-17.815	-20.944	-18.376 (↑ 2.568)
	CP-F	-11.929	-14.074	-12.540	**-16.099**	0.048	-	-11.136	-14.096	-11.920 (↑ 2.176)
RMC9	CP-T	-13.931	-15.894	-14.957	-15.829	-13.788	-6.438	-6.479	**-15.919**	-13.538 (↑ 2.381)
	CP-F	-11.410	-11.938	-9.515	**-12.605**	0.059	-	1.128	-11.917	-9.728 (↑ 2.189)
Average	CP-T	-15.675	**-18.523**	-16.896	-18.501	-16.146	-8.488	-12.147	-18.432	-15.957 (↑ 2.475)
	CP-F	-11.670	-13.006	-11.028	**-14.352**	0.054	-	-5.004	-13.007	-10.824 (↑ 2.183)

The table reports NMSE values in dB. CP-T and CP-F refer to the time-domain and frequency-domain channel prediction tasks, respectively. Values in **bold** indicate the best performance in each row.

Table S16 Zero-shot results for channel estimation using WiFo-2 pretrained on LH-CSI datasets without PKU-RM. A low prediction ratio and a 20 dB SNR are used.

		Full-shot			Zero-shot		
Dataset	Task	ChannelNet	Channelformer	LLM-based	Interpolation	WiFo-2	WiFo-2 (w/o RM)
RMF8	CE	-17.640	-16.365	-18.048	1.647	**-20.588**	-17.577 (↑ 3.011)
RMF9	CE	-14.456	-13.273	-15.368	1.702	**-15.994**	-14.233 (↑ 1.761)
Average	CE	-16.048	-14.819	-16.708	1.675	**-18.291**	-15.905 (↑ 2.386)

The table reports NMSE values in dB. CE refers to the channel estimation task. Values in **bold** indicate the best performance in each row.

Table S17 Variation in the prediction accuracy of WiFo-2 across different relative prediction horizons. A low prediction ratio and a 10 dB SNR are adopted.

Task	25%	50%	75%	100%
CP-T	-17.27	-16.03	-14.79	-13.63
CP-F	-16.44	-15.13	-13.90	-12.95

The table reports NMSE values in dB. CP-T and CP-F refer to the time-domain and frequency-domain channel prediction tasks, respectively.

default, this model features 1.47M parameters and is trained on 9,000 samples. Evaluated under an SNR of 10 dB and a 50% channel prediction ratio, the full-shot performance of the 3D ResNet yields an NMSE of -5.97 dB (Table S13). Notably, the WiFo-2 large version achieves a zero-shot NMSE of -10.22 dB on the same task, substantially outperforming the fully trained 3D ResNet.

First, we investigate the impact of model capacity by varying the parameter count from 1.17k to 113M while maintaining the default training set size (Table S18). We observe that the NMSE monotonically decreases as the parameter count

scales from 1.17k to 1.47M. However, further increases in model size yield diminishing returns, with performance effectively plateauing. Crucially, even when scaled to 113M parameters-surpassing the capacity of WiFo-2 large-the task-specific model's performance remains vastly inferior to WiFo-2's zero-shot capability. This demonstrates that model size is not the limiting bottleneck for task-specific architectures, thereby justifying our selection of the 1.47M configuration.

Second, we assess data scalability by varying the number of training samples across three orders of magnitude, from 0.9k to 900k, while keeping the default model size fixed (Table S19). Overall, the model performance scales with the training set size, as evidenced by the consistent decrease in NMSE. Nevertheless, even with an expanded dataset of 900k samples (corresponding to 14.7B data points), the model only achieves an NMSE of -7.13 dB, still falling well short of WiFo-2 large's zero-shot performance. This indicates that once the data volume reaches a critical threshold (our default 9k samples), expanding the dataset by two additional orders of magnitude yields a marginal gain of less than 1.2 dB in NMSE. Given the prohibitive overhead of data collection within a single scenario, establishing 9k samples as the full-shot benchmark is both practically justified and sufficient to reflect the saturated performance of a fully trained model under a localized system configuration.

Table S18 Frequency-domain channel prediction performance of 3D ResNet with different model parameters on the QC18 dataset, measured by NMSE in dB. The SNR is fixed at 10 dB, the prediction ratio is set to 50%, and the number of training samples is 9,000 by default.

Parameters	1.17k	131k	892k	1.47M (default)	15.3M	52.6M	113M
NMSE	-4.96 dB	-5.54 dB	-5.60 dB	-5.97 dB	-6.01 dB	-5.95 dB	-5.85 dB

Table S19 Frequency-domain channel prediction performance of 3D ResNet with different training samples on the QC18 dataset, measured by NMSE in dB. The SNR is fixed at 10 dB, the prediction ratio is set to 50%, and the model parameter is 1.47M by default.

Training samples	0.9k	4.5k	9k (default)	22.5k	45k	90k	450k	900k
CSI points	14.7M	73.7M	147M	369M	737M	1.47B	7.37B	14.7B
NMSE	-4.10 dB	-5.63 dB	-5.97 dB	-6.38 dB	-6.95 dB	-6.78 dB	-7.17 dB	-7.13 dB

To clearly disentangle the distinct sources of these performance gains, Table S20 provides a comprehensive comparison between the 3D ResNet baseline and two variants of WiFo-2. While 3D ResNet is optimized directly on the large-scale QC18 dataset, WiFo-2 undergoes pretraining on the LH-CSI dataset, which features highly heterogeneous CSI distributions. Remarkably, despite sharing parameter parity with 3D ResNet and being exposed to even fewer training CSI data points, the WiFo-2 little version delivers a zero-shot performance that eclipses the full-shot baseline of 3D

ResNet by 0.45 dB. This striking advantage underscores that WiFo-2's foundational superiority stems primarily from the synergistic integration of heterogeneous data, an advanced model architecture, and a robust pretraining paradigm. Building upon this structural foundation, the WiFo-2 large version unlocks an additional 2.64 dB gain solely by scaling up the model capacity.

Collectively, these empirical findings illuminate the underlying mechanisms driving WiFo-2's exceptional zero-shot capabilities. The primary catalyst is the combination of heterogeneous data and multi-task training, which equips the model with robust generalization capabilities to surpass fully trained, task-specific architectures in completely unseen scenarios. On top of this data-driven and architectural foundation, further expanding the model scale manifests a compounding benefit, capturing a substantial 2.64 dB performance leap and demonstrating the presence of distinct scaling laws in wireless foundation models.

Table S20 Performance comparison of WiFo-2 and 3D ResNet on the QC18 dataset. For WiFo-2, we provide the active/total parameter counts.

Model	Parameters	Training CSI points	NMSE
3D ResNet	1.5M	14.7B (QC18)	-7.13 dB
WiFo-2-little	1.1/2.2M	11.6B (LH-CSI)	-7.58 dB (Zero-shot)
WiFo-2-large	50.9M/100.9M	11.6B (LH-CSI)	-10.22 dB (Zero-shot)

10 Supplementary Note 10. Additional results for confidence prediction

Cumulative distribution function of the confidence prediction

Fig. S3 shows the error cumulative distribution function for the three tasks, with more than 80% of the samples having a prediction error below 5 dB. The average MAE for time-domain channel prediction, frequency-domain channel prediction, and channel estimation is 2.05 dB, 2.10 dB, and 2.51 dB, respectively. These results indicate that WiFo-2 provides accurate confidence estimates that inform model updates and network handovers, substantially enhancing reliability in real-world deployments.

Fine-tuning on zero-shot split of LH-CSI

In Fig. 2d of the main manuscript, the mean predicted NMSE is -17.87 dB, whereas the mean calculated NMSE is -16.72 dB, indicating that the confidence score is underestimated by 1.15 dB on average. This discrepancy is likely caused by the distribution shift between the LH-CSI pretraining split, on which confidence-enhanced pretraining was performed, and the LH-CSI zero-shot split, to which the pretrained confidence predictor was directly applied.

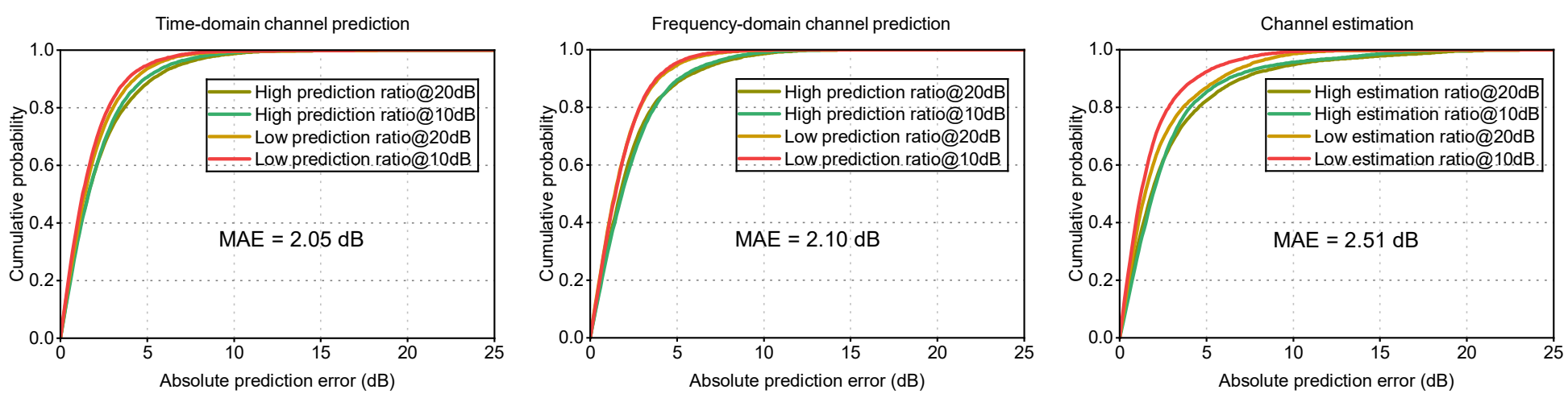

Fig. S3 The cumulative distribution function of the confidence prediction error across three tasks.

To mitigate this bias, we further calibrated the confidence output by few-shot fine-tuning on the LH-CSI zero-shot split. Specifically, we fine-tuned the model using 1% of the training data from each dataset in the LH-CSI zero-shot split and evaluated it on the corresponding test sets. Notably, during fine-tuning, we update only the learnable tokens related to confidence estimation and the lightweight confidence output head, leaving the remaining model parameters frozen. After fine-tuning, the mean predicted NMSE became -16.96 dB, reducing the bias relative to the calculated NMSE to 0.24 dB. We also observed that the confidence prediction became more accurate, with the MAE decreasing from 2.3 dB to 1.6 dB. The confidence prediction performance before and after fine-tuning across the three channel reconstruction tasks is visualized in Fig. S4. As shown, few-shot fine-tuning substantially reduces the distributional bias and yields more accurate confidence predictions. These results indicate that few-shot fine-tuning can further improve the reliability of confidence estimation on previously unseen datasets.

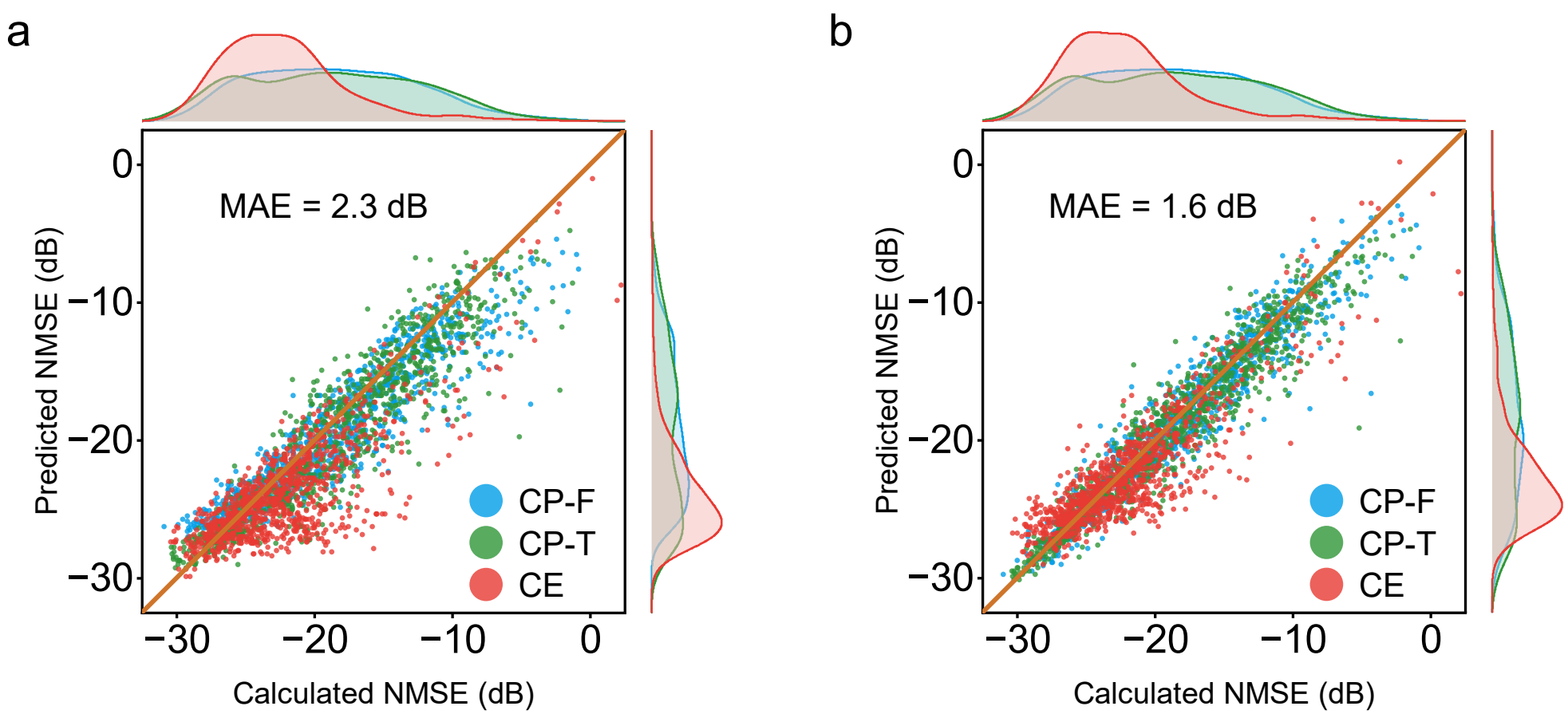

Fig. S4 Confidence prediction performance of WiFo-2 before and after fine-tuning across three channel reconstruction tasks. (a) Confidence outputs of WiFo-2 directly applied to unseen zero-shot split datasets. (b) Confidence outputs of WiFo-2 after fine-tuning with 1% of the samples

11 Supplementary Note 11. Additional results for CSI-related downstream tasks

Additional main results on CSI-related downstream tasks

Additional main experimental results for downstream tasks are presented in Fig. S5.

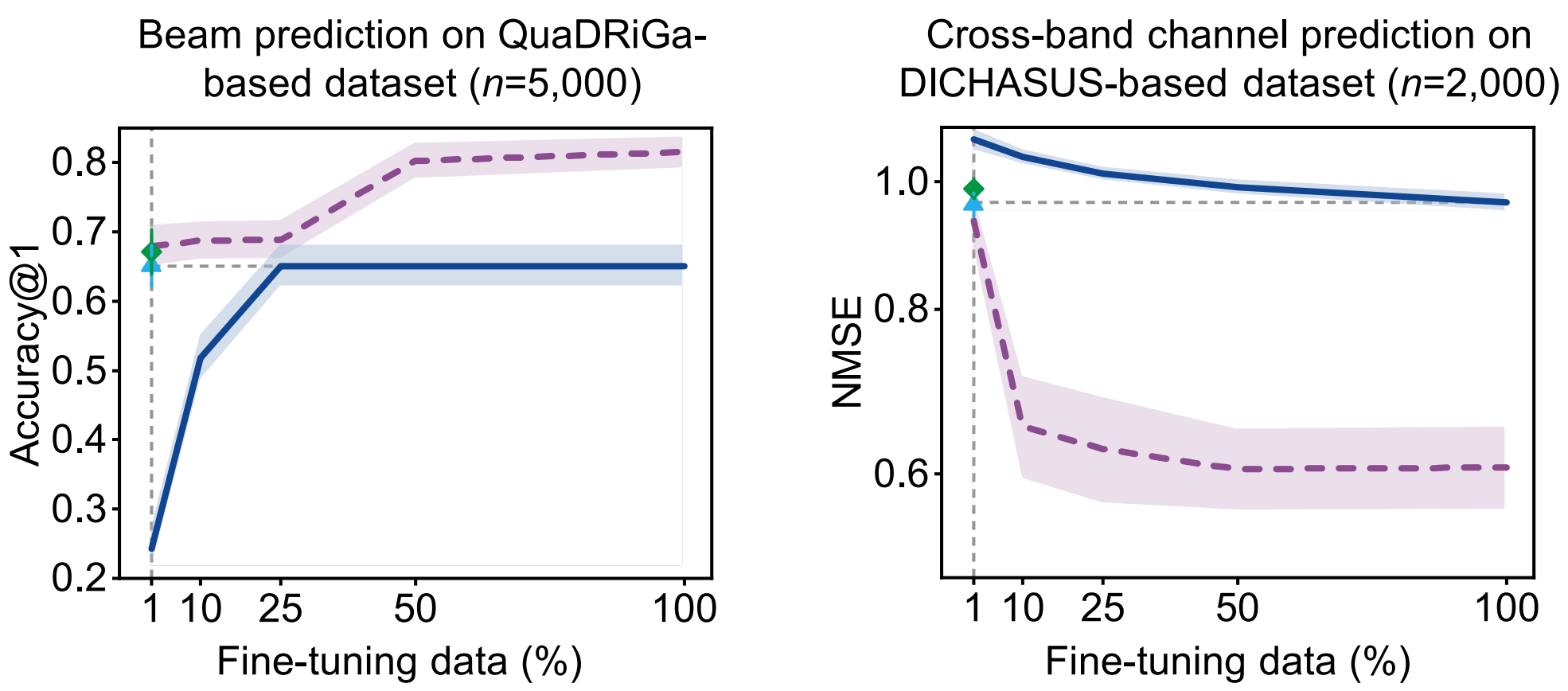

Fig. S5 Supplementary experimental results for beam prediction and cross-band channel prediction.

Ablations on vision-aided frequency-domain channel prediction

For the vision-aided channel prediction task, WiFo-2 shows a substantial advantage over task-specific methods. To further investigate the source of this gain, we added a modality ablation study, where we evaluate the zero-shot and few-shot performance of WiFo-2 under the CSI-only setting. The total number of training samples used for few-shot fine-tuning is 100. The results are shown in Table S21. Notably, even in the zero-shot setting, WiFo-2 still achieves an 8.17 dB performance gain over the full-shot performance of the task-specific model that uses both CSI and images. In addition, under few-shot fine-tuning, introducing additional image information brings an extra performance gain of approximately 0.75 dB to WiFo-2.

Overall, the main gain of WiFo-2 over task-specific AI models comes from its strong zero-shot channel prediction capability, while it also benefits from the incorporation of additional image information. These results demonstrate that the general representations learned by WiFo-2 enable effective cross-modal fusion and enhancement, thereby facilitating wireless system design assisted by multimodal information.

Table S21 Performance comparison of WiFo-2 and task-specific models for vision-aided frequency-domain channel prediction under different modality settings. NMSE (dB) is adopted as the evaluation metric. The number of training samples is 100.

Method	Modality	Fine-tuning data					
		0%	1%	10%	25%	50%	100%
Task-specific model	CSI+RGB	/	1.178	1.071	1.093	1.065	1.029
WiFo-2	CSI+RGB	0.157	0.108	0.097	0.082	0.065	0.065
WiFo-2	CSI	0.157	0.144	0.122	0.104	0.075	0.072

12 Supplementary Note 12. Visualization of the expert activation

Fig. S6 visualizes the distribution of expert activations across tasks and datasets, namely the fraction of tokens assigned to each expert of every CSI-SMoE layer. The SNR is set as 20 dB, and the low reconstruction ratio is considered. We observe that all experts are extensively utilized, and the activation patterns vary with both task and dataset. This demonstrates that the CSI-SMoE architecture effectively accommodates task and dataset heterogeneity, strengthening WiFo-2's generalization.

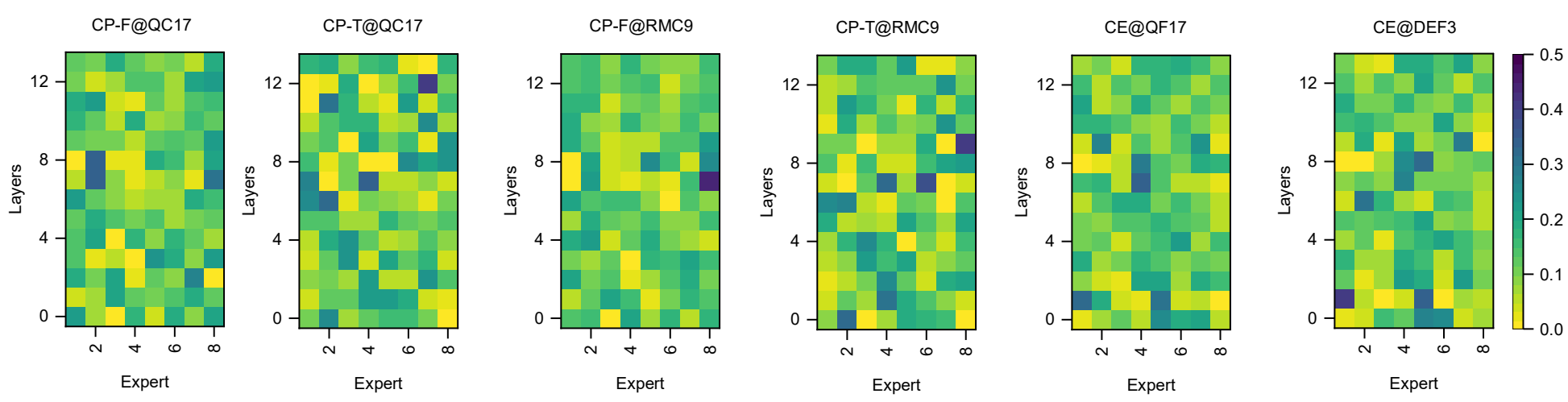

Fig. S6 The expert activation visualization of diverse channel reconstruction tasks and datasets.

13 Supplementary Note 13. Visualization of self-attention heat maps on different channel reconstruction tasks

Fig. S7 presents self-attention heat maps that visualize the regions WiFo-2 focuses on across different tasks. We derive visualizations from the first decoder layer's query–key dot product, treating the reference token's patch as the query and displaying its attended patches. The left image shows the real part of the time–frequency domain of the processed CSI, with the red box marking the region of the reference token. For time-domain and frequency-domain channel prediction, the network primarily attends

to tokens adjacent along the time or frequency axis, respectively. For channel estimation, the network focuses on surrounding tokens. These results indicate that WiFo-2 effectively captures task-adaptive time–frequency dependencies.

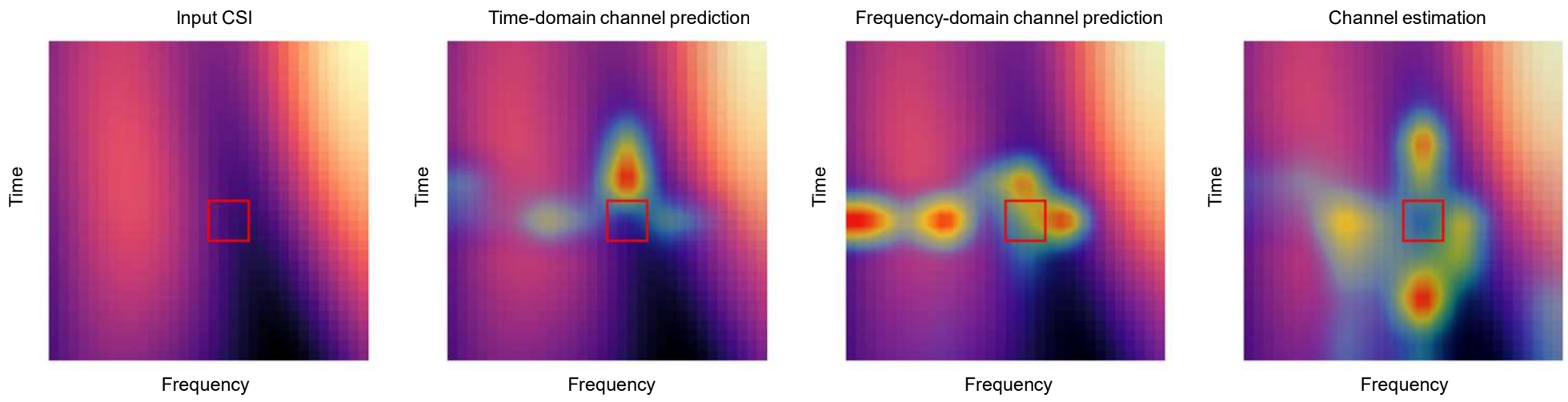

Fig. S7 Visualization of self-attention heat maps of WiFo-2 for different channel reconstruction tasks

14 Supplementary Note 14. Additional results for ablation studies

To assess the effectiveness of several key modules in the WiFo-2 design, we conduct an ablation study on the base version of WiFo-2 and evaluate performance both on zero-shot channel reconstruction and downstream tasks via fine-tuning. For zero-shot channel reconstruction, Table S22 reports the NMSE performance on three channel reconstruction tasks of high and low reconstruction ratios with the SNR fixed at 20 dB. For the downstream evaluation, we consider the wireless localization task with RMSE as the metric and the sub-6 GHz to mmWave beam prediction task with top-1 classification accuracy and SE as metrics, and the ablation results are shown in Table S23. It is observed that the version without any ablation achieves the best average performance on both the zero-shot channel reconstruction tasks and downstream tasks, which validates the effectiveness of the proposed key modules.

Replacing the proposed task-specific gating with a unified gate results in a significant performance degradation, including a 5.70 dB increase in the NMSE of zero-shot channel reconstruction, a 32.27% rise in the RMSE of wireless localization, and a 5.69% decrease in the accuracy of beam prediction. This clearly demonstrates the crucial role of the proposed task-specific gating in handling conflicts among tasks. Removing the random masking pretraining task leads to an overall degradation in performance, demonstrating that this task plays a key role in learning general CSI representations. In addition, removing the load balance loss likewise leads to a significant drop in performance, which can be attributed to an imbalance in expert assignment. Moreover, the dynamic ratios of masking and interpolation denoising used during pretraining are replaced with fixed values for ablation experiments. Specifically, the masking ratios of both the time- and frequency-masked reconstruction tasks are fixed at 25%, and the pilot-symbol placement proportions over (time, antenna, subcarrier) are fixed at (1/4, 1, 1/12) for the interpolation denoising tasks. It is observed that pretraining with fixed

ratios severely impairs generalization to unseen ratios and also leads to degraded performance on downstream tasks. Therefore, the proposed dynamic-ratio pretraining is essential for improving the foundation model's generalization across different ratios.

Table S22 Ablation results of WiFo-2 across three zero-shot channel reconstruction tasks. The SNR is fixed at 20 dB, and the reconstruction NMSE results in dB are reported.

	High ratio			Low ratio			
	CP-T	CP-F	CE	CP-T	CP-F	CE	Average
WiFo-2	**-8.809**	-9.254	**-13.027**	-12.271	-12.591	-16.818	**-12.128**
w/ unified gating	-5.433	-4.114	-3.165	-8.847	-7.465	-9.544	-6.428
w/o random masking	-8.709	**-9.306**	-12.289	-12.248	-12.469	-16.602	-11.937
w/o load balance loss	-8.553	-8.581	-12.805	-11.959	-12.287	-16.824	-11.835
w/o dynamic ratio	-3.558	-4.498	-2.567	**-12.619**	**-13.016**	**-16.860**	-8.853

Table S23 Ablation results of WiFo-2 on CSI-related downstream tasks, including wireless localization and sub-6 GHz to mmWave beam prediction.

	Wireless localization	Sub-6 GHz to mmWave beam prediction	
	RMSE (m)	Acc. @1	SE (bps/Hz)
WiFo-2	**1.156**	**84.4%**	**5.910**
w/ unified gating	1.529	79.6%	5.751
w/o random masking	1.324	81.3%	5.789
w/o load balance loss	1.313	82.8%	5.839
w/o dynamic ratio	1.399	81.6%	5.732

To assess the contribution of pretrained weights to WiFo-2's effectiveness on downstream tasks, Table S24 reports the results of an ablation study. Here, "scratch" denotes models trained from random initialization, and all models are fine-tuned using only 1% of the available downstream data. As observed, fine-tuning from pretrained weights consistently yields substantial improvements over training from scratch across all tasks, providing strong evidence for the effectiveness of the learned pretrained representations.

To validate the effectiveness of the proposed task-specific gating fine-tuning scheme, we compare it with the direct application of existing gating networks without fine-tuning. Relative to using existing gating networks, the additional fine-tuning overhead of the proposed gating network is nearly negligible. As shown in Fig. S8, the proposed gating fine-tuning scheme achieves SOTA performance across 8 tasks, confirming its ability to transfer general CSI knowledge to diverse CSI-related downstream tasks efficiently.

Table S24 Ablation study of pre-trained weights in WiFo-2 for downstream tasks.

Downstream Tasks	Dataset source	Metric	WiFo-2	Scratch
Scenario classification	QuaDRiGa	F1 score	**0.789**	0.409
Sub-6 GHz to mmWave beam prediction	QuaDRiGa	Acc.@1	**0.680**	0.651
		SE (bps/Hz)	**5.238**	5.130
Wireless localization	DICHASUS	RMSE (m)	**1.608**	1.864
Vision-aided frequency-domain channel prediction	SynthSoM	NMSE	**0.108**	1.378
		SE (bps/Hz)	**10.494**	5.100
CSI feedback	QuaDRiGa	NMSE	**0.128**	1.349
		SE (bps/Hz)	**11.510**	6.145
	Argos	NMSE	**0.922**	1.680
		SE (bps/Hz)	**9.235**	7.774
AoA estimation	DeepMIMO	MAE (rad)	**0.055**	0.572
Doppler estimation	QuaDRiGa	MAE (kHz)	**3.247**	3.379
Cross-band channel prediction	QuaDRiGa	NMSE	**0.431**	0.988
		SE (bps/Hz)	**9.038**	8.709
	DICHASUS	NMSE	**0.934**	4.743
		SE (bps/Hz)	**8.873**	6.446
Signal detection	QuaDRiGa	SER	**0.010**	0.274

Values in **bold** indicate the best performance in each row.

15 Supplementary Note 15. Additional results for scaling analysis

Scaling analysis [37] is crucial for guiding the design of wireless foundation models and the construction of corresponding datasets.

Scaling analysis on zero-shot channel reconstruction

Scaling analysis of zero-shot channel reconstruction with respect to dataset and model size has already been provided in Fig. 2e and f of the main manuscript. Here, we further clarify the composition of the pretraining datasets corresponding to the two curves in Fig. 2e.

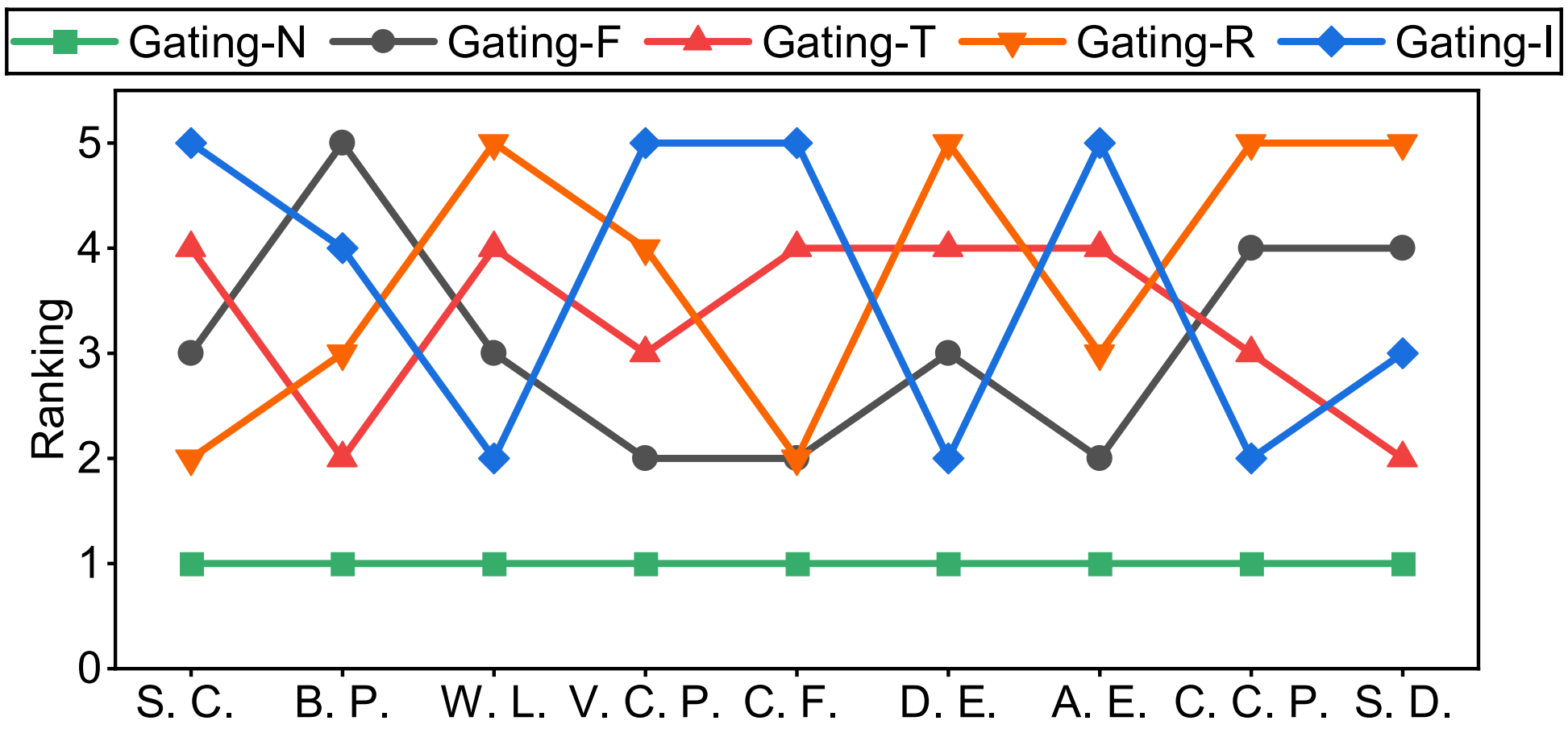

Fig. S8 Performance ranking of different gating schemes across eight tasks.

- **Varying heterogeneity**. We select five dataset configurations from the complete LH-CSI pretraining dataset, with each configuration containing a different number of sub-datasets. The components, number of sub-datasets, and pretraining tokens of each configuration are summarized in Table S25. In this setting, the five dataset configurations differ not only in the number of pretraining tokens, but also substantially in heterogeneity across system configurations, propagation scenarios, and other factors.
- **Invariant heterogeneity**. We also consider five dataset configurations, each covering all 63 sub-datasets but using different data proportions, namely 1, 1/2, 1/4, 1/8, and 1/16. The corresponding numbers of pretraining tokens are provided in Table S26. In this setting, the heterogeneity of the five datasets remains unchanged; that is, the training distribution is fixed, while only the dataset scale varies.

It can be observed that the degree of dataset heterogeneity has a pronounced impact on the scaling law. When additional training tokens are obtained by repeatedly sampling from a fixed set of sub-datasets, many samples share similar channel statistics and thus provide partially redundant information. In this case, performance improvement mainly comes from better fitting of already observed space-time-frequency patterns. In contrast, adding heterogeneous sub-datasets introduces new channel distributions associated with different scenarios, carrier frequencies, antenna configurations, mobility conditions, and acquisition modalities. These non-redundant samples expand the support of the pretraining distribution and expose the model to a broader range of propagation-induced CSI structures. Consequently, WiFo-2 learns more transferable representations, leading to a stronger scaling trend with respect to training tokens.

Table S25 Detailed description of the datasets under varying heterogeneity.

Number of sub-datasets	Components of sub-datasets	Pretraining tokens
63	QC1-QC16, QF1-QF16, VC, VF, LC, DC1, DC2, AC1-AC5, RMC1-RMC7, RMF1-RMF7, DEC1, DEC2, DEF1, DEF2, RTC1, RTC2	1.1×10^8
31	QC1-QC8, QF1-QF8, VC, VF, LC, DC1, AC1-AC2, RMC1-RMC3, RMF1-RMF3, DEC1, DEF1, RTC1	5.7×10^7
15	QC1-QC4, QF1-QF4, VC, VF, AC1, RMC1, RMF1, DEC1, DEF1	3.0×10^7
8	QC1-QC4, QF1-QF4	1.8×10^7
4	QC1, QC2, QF1, QF2	1.0×10^7

Table S26 Detailed description of the datasets under invariant heterogeneity.

Dataset proportion	Components of sub-datasets	Pretraining tokens
1	QC1-QC16, QF1-QF16, VC, VF, LC, DC1, DC2, AC1-AC5, RMC1-RMC7, RMF1-RMF7, DEC1, DEC2, DEF1, DEF2, RTC1, RTC2	1.1×10^8
1/2		5.5×10^7
1/4		2.8×10^7
1/8		1.4×10^7
1/16		6.9×10^6

Scaling analysis on CSI-related downstream tasks

Scaling analysis of CSI-related downstream tasks with respect to dataset and model size are illustrated in Table S27. For the 2nd, 4th, and 5th rows, the base-version WiFo-2 model is adopted while the number of sub-datasets in the pretraining corpus is varied. It can be observed that, as more sub-datasets are included, the performance on the downstream tasks improves significantly. This indicates that the constructed model can learn general CSI representations from large-scale heterogeneous datasets that are beneficial for generalization. For the 1st, 2nd, and 3rd rows, the full pretraining dataset is used, and the model size is scaled from small to large, where a notable performance improvement is observed. This can be attributed to the stronger modeling capacity of larger models. Consequently, WiFo-2 exhibits strong scalability with respect to both data volume and the number of model parameters. Even for relatively small models, further increasing the amount of pretraining data can still substantially enhance generalization performance, providing a meaningful path toward deploying wireless foundation models on computation-constrained communication devices.

16 Supplementary Note 16. Implementation details of the over-the-air experiments

The constructed wireless video transmission system adopts the 5G NR frame structure for downlink transmission, as illustrated in Fig. S9. Each frame has a duration of 10 ms and consists of 20 slots. Three types of slots are employed: synchronization signal

Table S27 Scaling results of WiFo-2 on CSI-related downstream tasks, including wireless localization and sub-6 GHz to mmWave beam prediction.

Model scale	Number of sub-datasets	Wireless localization	Sub-6 GHz to mmWave beam prediction	
		RMSE (m)	Acc. @1	SE (bps/Hz)
Small	63	1.236	79.6%	5.738
Base	63	1.156	84.4%	5.910
Large	63	**0.969**	**85.1%**	**5.955**
Base	31	1.174	83.6%	5.897
Base	15	1.264	82.0%	5.877

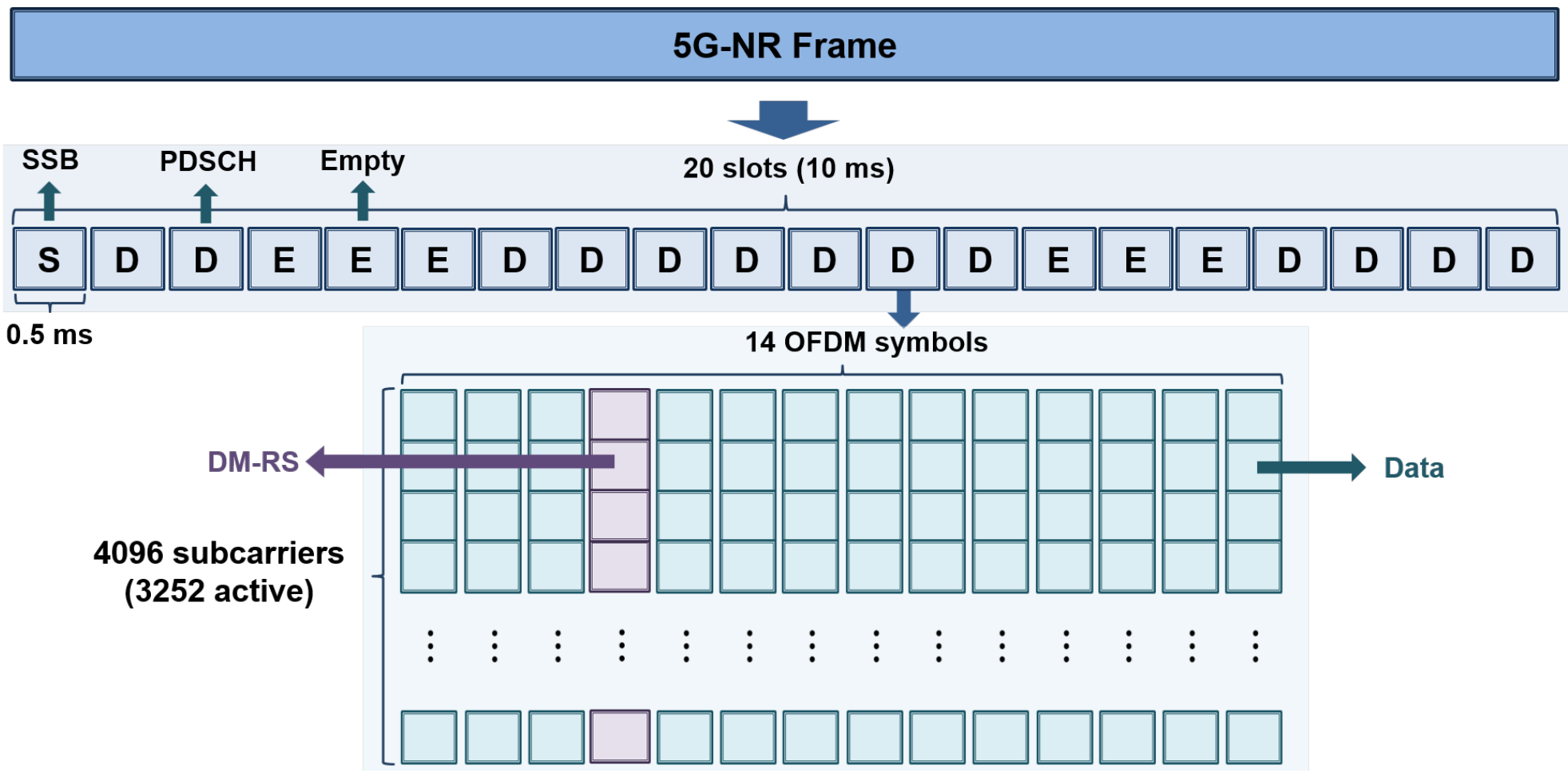

Fig. S9 An illustration of the adopted 5G NR frame structure.

block (SSB) slots for synchronization, physical downlink shared channel (PDSCH) slots for data transmission, and empty slots serving as guard intervals. Each PDSCH slot spans 14 OFDM symbols and 4096 subcarriers, of which 3252 consecutive subcarriers are activated, with a subcarrier spacing of 30 kHz. Within each PDSCH slot, one OFDM symbol is allocated to the demodulation reference signal (DM-RS) for channel estimation, while the remaining 13 OFDM symbols are used for data-bit transmission.

We process every 10 frames as one time–frequency unit, which introduces a buffering delay of 100 ms into the overall system. For model inference, we partition each unit into basic prediction or estimation blocks for parallel processing. For the channel prediction task, we construct 96 basic blocks, and within each basic block, the prediction ratio in the frequency domain is 25%. Specifically, in the frequency domain, the unit is divided into 48 groups, each consisting of 64 subcarriers with a subcarrier spacing of 120 kHz. In the time domain, the unit is divided into 2 groups, each consisting of 32

time instants with a sampling interval of 2.5 ms. For the channel estimation task, we construct 48 basic blocks and emulate sparse channel estimation with pilot densities of 1/8 and 1/24 in the time and frequency domains, respectively. Concretely, in the frequency domain, the unit is divided into 21 groups, each containing 72 subcarriers with a subcarrier spacing of 30 kHz. In the time domain, the unit is divided into 2 groups of 32 time instants with a sampling interval of 2.5 ms. For the scenario classification task, we determine the scenario type based on the CSI of basic estimation blocks. In addition, we repeat the time-frequency 2D CSI four times along the antenna dimension before feeding them into WiFo-2 for processing. Therefore, for the channel prediction and channel estimation tasks, the input sizes for a single inference pass are $(96, 2, 32, 4, 48)$ and $(48, 2, 32, 4, 72)$, respectively. The five dimensions correspond to the batch size, real and imaginary components, time, antennas, and subcarriers, respectively.

For quantized deployment, we specifically trained a dense-architecture WiFo-2 model comprising a four-layer encoder and a two-layer decoder, with a feature dimension of 512 and 13.29 M parameters. For the indoor sub-6 GHz scenario, we collected 1,000 CSI samples for fine-tuning, consisting of 500 basic channel prediction blocks and 500 basic channel estimation blocks. Using the same set of collected samples, we fine-tuned WiFo-2 and fully trained the MLP for comparison. To deploy WiFo-2 on the compute-constrained hardware prototype, we applied INT8 quantization using NVIDIA TensorRT. Specifically, we first converted WiFo-2 to the ONNX format, and then performed 8-bit quantization and calibration with TensorRT. Because ONNX export requires a fixed masking ratio, we generated separate ONNX models and TensorRT engines for channel estimation and channel prediction. The pre-quantization model achieves a per-token inference latency of 6.250 ms on an NVIDIA GeForce RTX 4090-24G GPU, while the quantized model attains an inference latency of 3.242 ms on the Jetson AGX Orin-64G edge device. As shown in Table S28, the NMSE changes in channel prediction and channel estimation are less than 0.1 dB after quantization. For the task-specific AI models, we employ a three-layer MLP with a hidden dimension of 64, comprising 6k parameters, which is likewise thoroughly trained on the 1,000 CSI samples. In addition, the first-order linear extrapolation is utilized for frequency-domain channel prediction, and the bilinear interpolation is adopted for channel estimation.

Table S28 Comparison of channel reconstruction NMSE before and after quantization of WiFo-2. The results for frequency-domain channel prediction and channel estimation are evaluated on the QC17 and QF17 datasets, respectively.

	Frequency-domain channel prediction	Channel estimation
FP32	-9.39 dB	-14.46 dB
INT8	-9.36 dB	-14.44 dB

We next report the practical deployment and inference overheads of WiFo-2, MLP, and interpolation for both channel estimation and channel prediction, enabling a

comprehensive comparison among different methods. The corresponding results are summarized in Tables S29 and S30. The metrics used in the comparison are defined as follows.

- **Precision**. Precision denotes the numerical precision used by a model or algorithm during practical deployment, such as INT8 or FP32. This metric characterizes the overhead associated with quantization and numerical representation on real hardware, and directly affects model storage, computational throughput, inference latency, and energy consumption. Owing to TensorRT quantization, WiFo-2 is converted from FP32 to the lower-precision INT8 format.
- **Arithmetic operations (GOPs)**. Arithmetic operations denote the theoretical number of operations required to complete one full channel estimation or channel prediction operation, measured in GOPs, where one GOP corresponds to 10^9 arithmetic operations. This metric quantifies the theoretical computational complexity of different methods.
- **Full-operation latency (ms)**. Full-operation latency denotes the end-to-end inference latency required to complete one full channel estimation or channel prediction operation after the model reaches stable execution, measured in milliseconds. This metric quantifies the additional latency introduced by inference.
- **Model storage (MB)**. Model storage denotes the amount of on-device storage required for deployment, measured in MB. This metric quantifies the static storage overhead of each method, namely the storage resources occupied by the deployed model or algorithm itself. For WiFo-2 and MLP, this includes the storage occupied by the deployed model parameters or TensorRT engine. For interpolation, because it contains no learnable parameters, the corresponding model storage overhead is treated as zero.
- **Peak memory footprint (MB)**. Peak memory footprint denotes the maximum device memory usage during one full inference operation, measured in MB. This metric characterizes the dynamic memory requirement during execution and indicates whether a method can run stably within the available memory budget of the target hardware platform.
- **Energy per operation (mJ)**. Energy per operation denotes the energy consumed to complete one full channel estimation or channel prediction operation, measured in mJ. This metric quantifies the energy consumption for single-operation inference across different methods in practical deployment.
- **Amortized average power (W)**. Amortized average power denotes the average power consumption obtained by amortizing the energy of a full operation over the system-level channel-processing period, measured in W. Specifically, it is computed as the energy per operation divided by the 100 ms inference period. This metric quantifies the average power increment introduced by periodic long-term execution.

Although WiFo-2 incurs higher deployment overheads than MLP and interpolation, its overheads remain well within the latency, storage, computational, memory, and power constraints of the designed hardware prototype. Specifically, the full-operation inference latencies of WiFo-2 are 31.94 ms and 21.48 ms for channel prediction and channel estimation, respectively. Because the prototype processes 10 frames as one

Table S29 Comparison of deployment overheads for channel prediction on the hardware prototype.

	Precision	Arithmetic operations (GOPs)	Full-operation latency (ms)	Model storage (MB)	Peak memory footprint (MB)	Energy per operation (mJ)	Amortized average power(W)
WiFo-2	INT8	267	31.94	26.8	765	504.47	5.04
MLP	FP32	0.403	7.17	0.0333	42.0	53.86	0.539
Interpolation	FP32	0.000811	1.83	0	0.309	20.30	0.203

Table S30 Comparison of deployment overheads for channel estimation on the hardware prototype.

	Precision	Arithmetic operations (GOPs)	Full-operation latency (ms)	Model storage (MB)	Peak memory footprint (MB)	Energy per operation (mJ)	Amortized average power(W)
WiFo-2	INT8	160	21.48	33.8	784	464.69	4.65
MLP	FP32	0.231	1.45	1.15	22.1	10.752	0.108
Interpolation	FP32	0.0133	1.20	0	0.0546	10.433	0.104

channel-processing unit, corresponding to a 100 ms processing period, these latencies fully satisfy the real-time latency requirement of the hardware prototype. In terms of model storage, the TensorRT engine of WiFo-2 occupies approximately 30 MB, whereas the Jetson AGX Orin provides 64 GB of eMMC storage. Therefore, the deployed WiFo-2 model imposes negligible pressure on the device storage. Regarding the peak memory footprint, WiFo-2 requires up to 784 MB of memory during inference, which is also far below the 64 GB memory capacity of the platform. In terms of power consumption, the Jetson AGX Orin used in our prototype supports a configurable power budget of up to 60 W. The inference power of WiFo-2 accounts for only approximately 8% of this budget, indicating a moderate workload on the current platform while still leaving sufficient power margin.

Finally, we further analyze the feasibility of deploying WiFo-2 on terminal-side edge chips. Considering the real-time channel estimation requirement in practical wireless systems, if each slot is used as the basic processing period and the slot duration is assumed to be 1 ms, the inference latency of the channel estimation algorithm should preferably be below 1 ms to meet the requirement of real-time baseband processing. We consider a typical terminal-side channel estimation configuration, where the transmitter and receiver are equipped with one and four antennas, respectively, and 1024 subcarriers and 12 OFDM symbols are processed within one slot. Accordingly, the input CSI tensor size can be represented as $(1, 2, 1024, 4, 12)$. Under this configuration, the arithmetic operations required by WiFo-2 for one full channel estimation operation are approximately 20 GOPs. The computation latency on a terminal-side chip can then be roughly estimated as

$$T_{\text{inf}} = \frac{\text{Ops}}{\eta P_{\text{peak}}}, \tag{16}$$

where Ops denotes the arithmetic operations required for one full inference operation, P_{peak} denotes the INT8 peak throughput of the target chip, and η denotes the effective hardware utilization. This utilization factor accounts for performance losses caused by operator mapping efficiency, on-chip and off-chip data access, pipeline stalls, control overheads, and preprocessing and postprocessing. Taking the AMD Versal AI Edge series adaptive SoC as a representative terminal-level programmable edge acceleration platform, the AI Engine INT8 peak throughput of its high-end model can reach 202 TOPS. Under a conservative assumption of 30% effective hardware utilization, the computation latency is estimated to be 0.33 ms. This estimated latency is below the 1 ms slot-level real-time processing constraint, suggesting that WiFo-2 has the potential to meet terminal-side real-time channel estimation requirements with INT8 quantization and hardware acceleration.

References

[1] Salihu, A., Rupp, M., Schwarz, S.: Self-Supervised and Invariant Representations for Wireless Localization. IEEE Trans. Wireless Commun. **23**(8), 8281–8296 (2024) https://doi.org/10.1109/TWC.2023.3348203

[2] Alikhani, S., Charan, G., Alkhateeb, A.: Large Wireless Model (LWM): A Foundation Model for Wireless Channels. arXiv (2024) 2411.08872

[3] Yang, T., Zhang, P., Zheng, M., Shi, Y., Jing, L., Huang, J., Li, N.: WirelessGPT: A Generative Pre-Trained Multi-Task Learning Framework for Wireless Communication. IEEE Netw. **39**(5), 58–65 (2025) https://doi.org/10.1109/MNET.2025.3579496

[4] Pan, G., Huang, K., Chen, H., Zhang, S., Häger, C., Wymeersch, H.: Large Wireless Localization Model (LWLM): A Foundation Model for Positioning in 6G Networks. arXiv (2025) 2505.10134

[5] Liu, B., Gao, S., Liu, X., Cheng, X., Yang, L.: WiFo: Wireless Foundation Model for Channel Prediction. Sci. China Inf. Sci. **68**, 162302 (2025) https://doi.org/10.1007/s11432-025-4349-0

[6] Catak, F.O., Kuzlu, M., Cali, U.: BERT4MIMO: A Foundation Model Using BERT Architecture for Massive MIMO Channel State Information Prediction. arXiv (2025) 2501.01802

[7] Guler, B., Geraci, G., Jafarkhani, H.: A Multi-Task Foundation Model for Wireless Channel Representation Using Contrastive and Masked Autoencoder Learning. IEEE J. Sel. Areas Commun. **44**, 4489–4504 (2026) https://doi.org/10.1109/JSAC.2026.3677157

[8] Liu, X., Gao, S., Liu, B., Cheng, X., Yang, L.: WiFo-CF: Wireless Foundation Model for CSI Feedback. IEEE Trans. Wireless Commun. **25**, 15039–15053 (2026) https://doi.org/10.1109/TWC.2026.3681311

[9] Zheng, T., Guo, J., Dai, L., Jin, S., Zhang, J.: Muse-fm: Multi-task environment-aware foundation model for wireless communications. arXiv (2025) 2509.01967

[10] Cui, Y., Guo, J., Wen, C.-K., Jin, S., Tong, E.: Leveraging Pre-Trained Large Language Models for CSI Feedback in Massive MIMO Systems. Authorea Prepr.

[11] Cheng, X., Liu, B., Liu, X., Liu, E., Huang, Z.: Foundation Model Empowered Synesthesia of Machines (SoM): AI-Native Intelligent Multi-Modal Sensing-Communication Integration. IEEE Trans. Netw. Sci. Eng. (2025) https://doi.org/10.1109/TNSE.2025.3587238 . Early Access

[12] Jaeckel, S., Raschkowski, L., Börner, K., Thiele, L.: QuaDRiGa: A 3-D Multi-Cell Channel Model With Time Evolution for Enabling Virtual Field Trials. IEEE

Trans. Antennas Propag. **62**(6), 3242–3256 (2014)

[13] Huang, Z., *et al.*: A Mixed-Bouncing Based Non-Stationarity and Consistency 6G V2V Channel Model With Continuously Arbitrary Trajectory. IEEE Trans. Wireless Commun. **23**(2), 1634–1650 (2023)

[14] Yaman, I., *et al.*: The LuViRA Dataset: Synchronized Vision, Radio, and Audio Sensors for Indoor Localization. In: 2024 IEEE International Conference on Robotics and Automation (ICRA), pp. 11920–11926 (2024)

[15] Euchner, F., Gauger, M., Dörner, S., Brink, S.: A Distributed Massive MIMO Channel Sounder for "Big CSI Data"-Driven Machine Learning. In: WSA 2021; 25th International ITG Workshop on Smart Antennas, pp. 1–6 (2021)

[16] Shepard, C., Ding, J., Guerra, R.E., Zhong, L.: Understanding Real Many-Antenna MU-MIMO Channels. In: 2016 50th Asilomar Conference on Signals, Systems and Computers, pp. 461–467 (2016)

[17] Alkhateeb, A.: DeepMIMO: A Generic Deep Learning Dataset for Millimeter Wave and Massive MIMO Applications. arXiv (2019) 1902.06435

[18] Hoydis, J., Cammerer, S., Ait Aoudia, F., Nimier-David, M., Maggi, L., Marcus, G., Vem, A., Keller, A.: Sionna. https://nvlabs.github.io/sionna/ (2022)

[19] Jiang, H., Cui, M., Ng, D.W.K., Dai, L.: Accurate Channel Prediction Based on Transformer: Making Mobility Negligible. IEEE J. Sel. Areas Commun. **40**(9), 2717–2732 (2022)

[20] Feichtenhofer, C., Fan, H., Malik, J., He, K.: SlowFast Networks for Video Recognition. In: Proceedings of the IEEE/CVF International Conference on Computer Vision, pp. 6202–6211 (2019)

[21] Jiang, W., Schotten, H.D.: Deep Learning for Fading Channel Prediction. IEEE Open J. Commun. Soc. **1**, 320–332 (2020)

[22] Liu, B., Liu, X., Gao, S., Cheng, X., Yang, L.: LLM4CP: Adapting Large Language Models for Channel Prediction. J. Commun. Inf. Netw. **9**(2), 113–125 (2024)

[23] Yin, H., Wang, H., Liu, Y., Gesbert, D.: Addressing the Curse of Mobility in Massive MIMO With Prony-Based Angular-Delay Domain Channel Predictions. IEEE J. Sel. Areas Commun. **38**(12), 2903–2917 (2020)

[24] Xiaoming, S., Shiyu, W., Yuqi, N., Dianqi, L., Zhou, Y., Qingsong, W., Jin, M.: Time-MoE: Billion-Scale Time Series Foundation Models With Mixture of Experts. In: ICLR 2025: The Thirteenth International Conference on Learning Representations (2025). International Conference on Learning Representations

[25] Soltani, M., Pourahmadi, V., Mirzaei, A., Sheikhzadeh, H.: Deep Learning-Based Channel Estimation. IEEE Commun. Lett. **23**(4), 652–655 (2019)

[26] Luan, D., Thompson, J.S.: Channelformer: Attention-Based Neural Solution for Wireless Channel Estimation and Effective Online Training. IEEE Trans. Wireless Commun. **22**(10), 6562–6577 (2023)

[27] Sun, Z., Wang, K., Sun, R., Chen, Z.: Channel State Identification in Complex Indoor Environments With ST-CNN and Transfer Learning. IEEE Commun. Lett. **27**(2), 546–550 (2023)

[28] Alrabeiah, M., Alkhateeb, A.: Deep Learning for mmWave Beam and Blockage Prediction Using Sub-6 GHz Channels. IEEE Trans. Commun. **68**(9), 5504–5518 (2020)

[29] Salihu, A., Schwarz, S., Rupp, M.: Attention Aided CSI Wireless Localization. In: 2022 IEEE 23rd International Workshop on Signal Processing Advances in Wireless Communications (SPAWC), pp. 1–5 (2022)

[30] Wen, C.-K., Shih, W.-T., Jin, S.: Deep Learning for Massive MIMO CSI Feedback. IEEE Wireless Commun. Lett. **7**(5), 748–751 (2018) https://doi.org/10.1109/LWC.2018.2818160

[31] Cui, Y., Guo, A., Song, C.: TransNet: Full Attention Network for CSI Feedback in FDD Massive MIMO System. IEEE Wireless Commun. Lett. **11**(5), 903–907 (2022)

[32] Ahmadi, H., Kuhestani, A., Keshavarzi, M.R., Mesin, L.: Semantic-Aware Doppler Shift Estimation for Low Earth Orbit Satellite Communications. TechRxiv (2025) https://doi.org/10.36227/techrxiv.174495689.97257048/v2 . Preprint

[33] Cheng, X., *et al.*: SynthSoM: A Synthetic Intelligent Multi-Modal Sensing-Communication Dataset for Synesthesia of Machines (SoM). Sci. Data **12**, 819 (2025) https://doi.org/10.1038/s41597-025-05065-x

[34] He, K., Zhang, X., Ren, S., Sun, J.: Deep Residual Learning for Image Recognition. In: Proc. IEEE/CVF Conf. Comput. Vis. Pattern Recognit. (CVPR), pp. 770–778 (2016)

[35] Nam, Y., Choi, J.: Multi-Modal Variable-Rate CSI Reconstruction for FDD Massive MIMO Systems. arXiv (2025) 2501.11926

[36] He, H., Wen, C.-K., Jin, S., Li, G.Y.: Model-Driven Deep Learning for MIMO Detection. IEEE Trans. Signal Process. **68**, 1702–1715 (2020)

[37] Kaplan, J., et al.: Scaling Laws for Neural Language Models. arXiv (2020) 2001.08361
